# Comprehensive Identification of Drought Tolerance QTL-Allele and Candidate Gene Systems in Chinese Cultivated Soybean Population

**DOI:** 10.3390/ijms21144830

**Published:** 2020-07-08

**Authors:** Wubin Wang, Bin Zhou, Jianbo He, Jinming Zhao, Cheng Liu, Xianlian Chen, Guangnan Xing, Shouyi Chen, Han Xing, Junyi Gai

**Affiliations:** 1 Soybean Research Institute, Nanjing Agricultural University, Nanjing 210095, China; wangbean@njau.edu.cn (W.W.); 18756019871@139.com (B.Z.); hjbxyz@gmail.com (J.H.); jmz3000@126.com (J.Z.); wildsoybean@163.com (C.L.); cxl005200@163.com (X.C.); xinggn@njau.edu.cn (G.X.); hanx@njau.edu.cn (H.X.); 2 MOA National Center for Soybean Improvement, Nanjing Agricultural University, Nanjing 210095, China; 3 MOA Key Laboratory of Biology and Genetic Improvement of Soybean (General), Nanjing Agricultural University, Nanjing 210095, China; 4 State Key Laboratory for Crop Genetics and Germplasm Enhancement, Nanjing Agricultural University, Nanjing 210095, China; 5 Jiangsu Collaborative Innovation Center for Modern Crop Production, Nanjing Agricultural University, Nanjing 210095, China; 6 Institute of Genetics and Developmental Biology, Chinese Academy of Sciences, Beijing 100101, China; sychen@genetics.ac.cn

**Keywords:** soybean (*Glycine max* (L.) Merr.), drought tolerance (DT), membership index value of relative plant weight (MPW), membership index value of relative plant height (MPH), restricted two-stage multi-locus multi-allele genome-wide association study (RTM-GWAS), QTL-allele matrix, quantitative real-time PCR (qRT-PCR)

## Abstract

Drought is one of the most important factors affecting plant growth and productivity. The previous results on drought tolerance (DT) genetic system in soybean indicated a complex of genes not only few ones were involved in the trait. This study is featured with a relatively thorough identification of QTL-allele/candidate-gene system using an efficient restricted two-stage multi-locus multi-allele genome-wide association study, on two comprehensive DT indicators, membership index values of relative plant weight (MPW) and height (MPH), instead of a single biological characteristic, in a large sample (564 accessions) of the Chinese cultivated soybean population (CCSP). Based on 24,694 multi-allele markers, 75 and 64 QTL with 261 and 207 alleles (2–12/locus) were detected for MPW and MPH, explaining 54.7% and 47.1% of phenotypic variance, respectively. The detected QTL-alleles were organized into a QTL-allele matrix for each indicator, indicating DT is a super-trait conferred by two (even more) QTL-allele systems of sub-traits. Each CCSP matrix was separated into landrace (LR) and released cultivar (RC) sub-matrices, which showed significant differentiation in QTL-allele constitutions, with 58 LR alleles excluded and 16 new ones emerged in RC. Using the matrices, optimal crosses with great DT transgressive recombinants were predicted. From the detected QTL, 177 candidate genes were annotated and validated with quantitative Real-time PCR, and grouped into nine categories, with ABA and stress responders as the major parts. The key point of the above results is the establishment of relatively full QTL-allele matrices composed of numerous gene functions jointly conferring DT, therefore, demonstrates the complexity of DT genetic system and potential of CCSP in DT breeding.

## 1. Introduction

Approximately 41% of the world land surface is dryland [1], and even in humid and semi-humid areas, the abrupt climate changes, including increased droughts, are found throughout much of the world [2,3]. As such, drought has been, and will continue to be, one of the most important factors affecting plant growth and productivity [4,5]. For example, Texas in the US experienced a driest and warmest 12-month period of drought in 2010–2011 [6], which resulted in an estimated US7.5 billion dollars in agricultural losses [3]. According to China’s agricultural statistics, about 70–80 billion kilograms of food crop are lost due to droughts each year, which accounts for 17% of the total national production [7].

To evaluate the drought tolerance (DT) of plants, different indicators, such as water use efficiency [8,9], leaf hydraulic conductance [10], leaf water status traits [11], leaf δ^13^C [12] and so on, have been used, but each of these indicators may involve only individual biological process, while in crops, DT related to the final growth (products), should be a comprehensive trait resulted from a series of biological processes, which are genetically controlled by a series of genes [13]. Thus, the indicator should represent the overall perspective of DT. The growth traits, such as relative values of plant weight and plant height, are considered as comprehensive DT indicators because all the individual DT biological processes will be finally reflected on plant growth and its end products [14,15,16].

Soybean (*Glycine max* (L.) Merr.), a miracle crop rich in protein and oil originated in China, has been cultivated for approximately five thousand years [17,18]. In the long term of domestication and improvement, there have been accumulated a great number of genetic variations for all kinds of traits, including DT, in the landraces and therefore released cultivars. These historical materials compose the current germplasm population, and in fact, a gene reservoir for modern soybean breeding [19,20].

For effective utilization of germplasm in plant breeding, the basic step is to explore the population’s genetic constitution, including the QTL (quantitative trait loci) or genes with their corresponding alleles. Linkage mapping has provided a first way to detect the genes and their alleles, but can only detect QTL/genes polymorphic between the two parents in a cross, therefore, the previous DT QTL detection was limited to few crosses or parental materials. Genome-wide association study (GWAS) is a potential way to detect whole-genome QTL in a natural population with the advantages of high mapping resolution, multiple alleles per locus, a large source population and less time consumption [21]. This approach has been applied to dissect the genetic base of various traits in maize [22], rice [23], Arabidopsis [24], soybean [25] and other plants. In the previous GWAS using SNPs (single nucleotide polymorphisms) as markers, only two alleles on each locus could be detected, which did not match the property of multiple alleles in a natural population. Meanwhile, inbreeding is usually involved in plant species, especially in the self-pollinated species of soybean, which may cause population structure bias along with admixture, resulting in a large number of false positives. Different approaches have been suggested to correct the population bias [26], accompanied with a conservative significance level, such as a Bonferroni correction. As a result, only a handful of QTL were identified, accounting for only a relatively small part of the phenotypic variation [27,28]. This result could not match the requirement for a thorough exploration of the entire QTL-allele system in plant breeding and population genetic studies.

To raise the GWAS power and solve the missing and overflowing heritability problems, He et al. [29] designed a restricted two-stage multi-locus multi-allele GWAS (RTM-GWAS) procedure for plant species. Two innovations are involved in RTM-GWAS. One is to organize sequential SNPs into SNPLDBs (SNP linkage disequilibrium blocks) as genomic markers with multiple alleles, accordingly, using eigenvectors of the SNPLDB genetic-similarity matrix to match the untraceable comprehensive population structure bias. The other is to take a two-stage strategy with a single-locus model pre-selection of markers followed by a multi-locus multi-allele model stepwise regression for QTL identification under a reasonable experiment-wise significance level for all QTL (rather than Bonferroni correction as experiment-wise significance level for single-locus model) to control overflowing or missing heritability. By using RTM-GWAS in the soybean germplasm populations, the detected QTL systems could explain 72.2% and even up to 98.2% phenotypic variation for isoflavone content and 100-seed weight by Meng et al. [25] and He et al. [29], respectively. In our group, Khan et al. [30] reported that the DT QTL system could explain 88.6–95.9% phenotypic variation by using RTM-GWAS in a nested association mapping population tested under polyethylene-glycol (PEG) treatment using relative shoot and root lengths as indicators, but only three parental materials were involved. Based on Khan et al. [30], we supposed to extend the study to the Chinese cultivated soybean population (CCSP).

The present study aimed at to explore the genome-wide QTL conferring DT in the CCSP, to identify the evolutionary changes of QTL-allele structure from landrace subpopulation (LRS) to released cultivar subpopulation (RCS), to predict the DT genetic improvement potential in the CCSP, and to infer the DT candidate gene system through annotation and qRT-PCR (quantitative real-time polymerase-chain-reaction) verification. The study is featuring using QTL-allele matrices of comprehensive DT traits, relative plant weight and plant height to show the comprehensive genetic structure of the CCSP and the population evolutionary changes from LRS to RCS.

## 2. Results

### 2.1. Wide Variation of Drought Tolerance in the CCSP

In the CCSP, two DT indicators, MPW and MPH, represented membership index value of relative plant weight and plant height under water stress to non-stress condition, respectively, showing wide phenotypic variation with ranges of −0.362–1.411 and −1.323–1.792. The values beyond 1.000 or below 0.000 means that the materials were more drought-tolerant or more drought-sensitive than the corresponding checks, respectively (Table 1). The heritability value of MPW was 81.3%, higher than 76.0% of MPH. The value of correlation coefficient between MPW and MPH was 0.55, which was significant but not high, indicating the genetic systems of the two indicators might be different. According to the values of MPW and MPH, 12 highly tolerant and 12 highly sensitive accessions were identified (Table S1), including the five tolerant and five sensitive checks and additional seven highly tolerant and seven highly sensitive accessions. The broad variation further called our attention to explore the genetic constitution of DT in the CCSP.

The means and ranges of the two indicators for the whole population and the landrace (LRS) and released cultivar subpopulations (RCS) showed a similar result. The mean value of DT for RCS was some less than that of LRS, which indicates that the breeding for DT had not been emphasized in previous breeding programs although the former was developed from the latter (Table 1).

### 2.2. QTL-Allele System of Drought Tolerance in the CCSP

**Figure 1:**
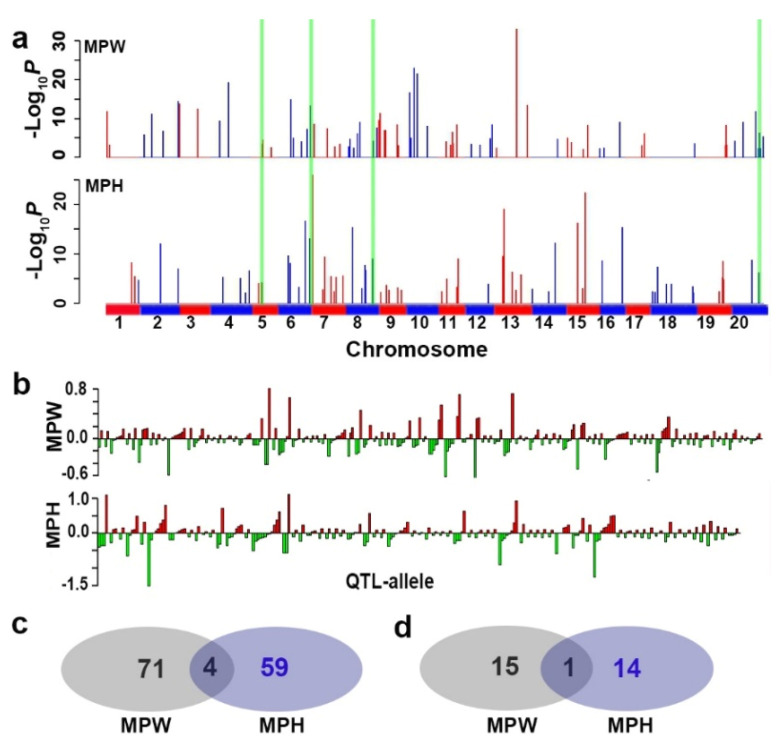
The detected QTL conferring two drought tolerance indicators. (**a**) The detected drought tolerance QTL for the two indicators located on chromosomes over the whole genome. The probability −Log_10_*^P^* value indicates the significance level of the corresponding QTL. The vertical green bars spanning multiple graphs denote the four shared QTL/markers between the two indicators. (**b**) The allele effects of the DT QTL for MPW and MPH; the red and green bars indicate the positive and negative alleles, respectively. (**c**) and (**d**), the Venn-Diagrams of the whole and LC-major QTL for the two indicators, respectively.

Using the 24,694 SNPLDBs in the RTM-GWAS procedure, at the first stage, 7795 and 7382 SNPLDBs were preselected for the second stage analysis, and then 75 and 64 QTL were detected for MPW and MPH, with −Log_10_*^P^* values ranging in 2.2~33.0 and 2.2~25.9, respectively (Table 2 and Table 3, Figure 1). The genetic contribution (*R*^2^) of individual QTL ranged from 0.1% to 3.5%, with Gm13_BLOCK338 and Gm07_BLOCK25 having the highest significance values (−Log_10_*^P^*) for respective indicators (Figure 1a, Table 2 and Table 3). Among these loci, 16 and 14 ones were the large-contribution major QTL (LC-major QTL) with *R*^2^ values more than 1.0% (Table 2 and Table 3). 

On the 75 and 64 loci for MPW and MPH, 261 and 207 alleles were detected with 2~12 ones per locus, and among these alleles, 127 and 106 had positive effects, and 134 and 101 had negative effects, respectively (Figure 1b, Table 4).

Based on the RTM-GWAS results, the composition of DT QTL system in CCSP was explored (Table 4). For MPW, 81.3% (heritability value) of the phenotypic variation was explained by genetic variation, in which the total *R^2^* of 16 LC-major QTL and 59 SC-major QTL were 25.9% and 28.8%, respectively, in a total of 75 detected QTL explaining 54.7% of phenotypic variation, and the remained 26.6% phenotypic variation might be explained by the collective unmapped minor QTL. The genetic structure of MPH was similar to that of MPW. In a total, 135 QTL/markers were detected for the two DT indicators, among which only 4 QTL/markers were shared between the two indicators (Figure 1a,c). The shared QTL only explained a small part of phenotypic variation with values of 2.8% and 3.4%, respectively, not very much, but contained two LC-major QTL/SNPLDBs, Gm06_BLOCK576 and Gm08_BLOCK466, which might be the most important QTL for DT in soybean. The above results make us understand that DT is a complex trait, different indicators have their own genetic systems, and all the detected 135 QTL/markers are members of the DT genetic system. Therefore, in the following text, they will be considered as a joint genetic system conferring a super-DT-trait.

### 2.3. MPW and MPH QTL-ALLELE MATRICES as a Compact Genetic DT Structure of CCSP

All the detected QTL-alleles with their effects of the 564 accessions for each indicator were organized into a QTL-allele matrix, which was a compact form of the genetic structure of CCSP as well as that of each accession. 

**Figure 2:**
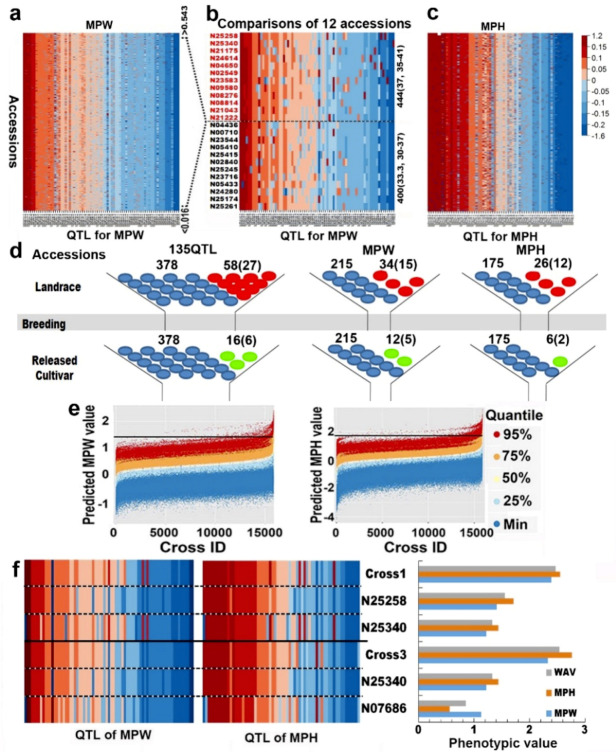
The DT QTL-allele matrices of CCSP and their application in population differentiation, optimal cross design. (**a**) and (**c**), The QTL-allele matrices of MPW and MPH, with the vertical axis for accessions in an increasing tendency from bottom to top, and the horizontal axis for QTL with the number of positive alleles increasing from right to left. The warm color indicates a positive allele and the cool color indicates a negative allele with the depth of color indicating the degrees of allele effect. (**b**) The MPW QTL-allele matrix of the 12 high-DT accessions (red letters) and 12 low-DT checks (black letters). The data at the right side of the matrix is the total number of positive alleles (outside of the parentheses) and the average number of positive alleles followed by a range (in parentheses). The elite accessions have more positive alleles. (**d**) Changes of the genetic diversity from the landrace subpopulation (LRS) to the released cultivar subpopulation (RCS). The colored dots represent different DT alleles, including the retained alleles in blue color from LRS to RCS, lost alleles in red color during the processes of improvement, and newly emerged alleles in green in RCS. The number in parentheses represents the number of positive alleles. The shaded areas indicate bottleneck effect had happened. (**e**) The distribution of predicted MPW and MPH of progenies from possible crosses. On the horizontal axis, the crosses are arranged according to the predicted means in ascending order from the left to the right. The vertical axis represents the predicted phenotypic values of the predicted progenies. The scattered dots in different colors represent the different quantiles of 10,000 progenies. The black horizontal line indicates the maximum value of MPW (1.411), and MPH (1.792) in the CCSP. (**f**) The phenotype value and genetic structure of the two crosses along with their parents. WAV means the weighted average value over the two indicators.

Figure 2a,c show the QTL-allele matrices in colors for the two indicators. The matrices showed that all the accessions contained both positive and negative alleles, indicating a great recombination potential for breakthrough segregants in the population. The number of positive alleles were increased with the increase of indicator value, which explained why an accession performed well in DT. For example, 444 positive alleles were contained in the 12 highly tolerant accessions (MPW > 0.545) with an average of 37 positive alleles per accession (ranging in 35~41), but only 400 positive alleles in the 12 highly sensitive accessions (MPW < 0.016) with an average of 33.3 per accession (ranging in 30~37). The difference between the two groups was essentially due to the difference in allele effects (Figure 2b). It was a novel way in population genetics to use QTL-allele matrix based on a relatively thorough QTL-allele identification to represent the germplasm population structure.

### 2.4. Population Genetic Differentiation from Landraces to Released Cultivars

The CCSP QTL-allele matrix was separated into its components, LRS and RCS. The independence of the allele frequency distribution of detected QTL between LRS and RCS was tested with Chi-square criterion, and 87 of the 135 QTL showed significant differentiation at *p* = 0.05 with an average of 4.4 QTL per chromosome, ranging from 1 on Gm05 and Gm17 to 8 on Gm08 (Table S3). The genetic differentiation performed mainly as different frequency distribution between LRS and RCS, especially on the 43 loci listed in Table 5. For MPW, 27 out of 75 loci (36.0%) were with allele changes, and out of 261 (134 negative plus 127 positive) alleles, 34 (19 negative, 15 positive) LRS alleles were excluded but 12 (7 negative, 5 positive) alleles were newly emerged in RCS, and in a total, 46 (26 negative, 20 positive) alleles were changed on the 27 loci (Figure 2d, Table 5). For MPH, 19 out of 64 loci (29.7%) were with allele changes, and out of 207 (101, 106) alleles, 26 (14, 12) LRS alleles were excluded but 6 (4, 2) alleles emerged in RCS, and in a total, 32 (18, 14) alleles were changed. Among the 43 loci with allele changes, Gm06_BLOCK576, Gm08_BLOCK466 and GM20_39658089 were joint ones shared between MPW and MPH (Table 5). Altogether, for the supper-DT-trait composed of MPW and MPH, there were 436 (217 negative, 219 positive) alleles on 135 DT QTL in the LRS, from which 378 (186, 192) alleles on 135 DT loci passed to RCS, but 58 (31 negative, 27 positive) alleles on 36 loci did not appear in RCS or excluded during the breeding processes (Figure 2d, Table 5). However, 16 (10 negative, 6 positive) new alleles on 13 loci were emerged during the breeding processes in the RCS. Among the 58 disappeared alleles and the 16 emerged alleles, both positive and negative effect alleles were involved, with 27 negative alleles vs. 31 positive alleles in excluded ones and 10 negative alleles vs. 6 positive alleles in emerged ones, in a total of 41 negative vs. 33 positive in a total of 74 changed alleles. Thus, in the excluded and emerged alleles, the number of negative alleles and number of positive alleles were roughly about similar, the allele changes from LRS to RCS was not obviously orientation-directed. The significant genetic differentiation between the LRS and RCS at the subpopulation and individual locus level caused the DT reduction from LRS to RCS, from 0.434 to 0.401 for MPW and from 0.150 to 0.082 for MPH, which suggested that the QTL-allele structure changes from LRS to RCS caused the subpopulation mean DT values changed. However, both the facts of the small phenotypic DT reduction and non-orientation-directed genetic differentiation from LRS to RCS implied that DT breeding did not receive enough attention in previous cultivar development, therefore, should be enhanced in the future in China.

In addition, among the 43 loci with allele changing, there appeared very active loci, Gm06_ BLOCK491for MPH with five alleles excluded in RCS; Gm15_BLOCK409 for MPW with three alleles emerged and one allele excluded in RCS; and Gm17_BLOCK344 for MPW with 4 alleles excluded in RCS. Among the newly emerged alleles in RCS, the allele (a3) on Gm06_BLOCK576 was associated with high positive effects for both MPW and MPH, while among the specific alleles in LRS (absented in RCS), the alleles a1 and a2 on Gm08_BLOCK466 were with negative effects for both MPW and MPH (Figure 2d, Table 5). These specific loci-alleles should be potential in their gene functions.

### 2.5. Prediction of Optimal Cross for Drought Tolerance Improvement

Based on the QTL-allele matrices, the optimal crosses of DT were predicted. Figure 2e showed the distributions of predicted MPW and MPH values for the simulated progenies. There were 3319 and 3214 crosses with the predicted 95th percentile values exceeding the maximum phenotypic value in the CCSP for the respective indicators, among them, 745 crosses were jointly superior for the two indicators. The best top 10 predicted crosses were listed in Table 6, among which the parental phenotypic values of MPW and MPH ranged in 0.645~1.411 and 0.059~1.712, respectively, while the predicted 95th percentile values of progenies ranged as 2.107~2.392 and 2.244~3.135, indicating that a great transgression might be obtained from these crosses. As shown in Table 6, the two parents of Cross 1 (N25340 × N25258) both had high values for the two indicators, and the two parents of Cross 8 (N24359 × N25340) had medium and high values for the two indicators. However, both crosses could produce elite progenies with 95th percentile values up to 2.392 and 2.140, 2.552 and 2.417, 2.460 and 2.274 for MPW, MPH and WAV (weighted average value of the two indicators), respectively. The high × medium crosses (Cross 2 and 3) even can provide better segregants than the high × high cross (Cross 1), because more elite alleles could be converged in the former cases (Figure 2f and Table 6). 

In conventional breeding, breeders usually used high × high strategy for designing crosses, while the present results implied that in marker-assisted breeding, the parental selection may extend to a broader range, which makes the breeders have more freedom in breeding by design. In summary, the marker-assisted cross design based on the QTL-allele matrix can take the advantage of converging the best alleles and therefore provide a way to create innovative materials with the desired genetic structure.

### 2.6. The Candidate Gene System of Drought Tolerance Inferred from Detected QTL

**Figure 3:**
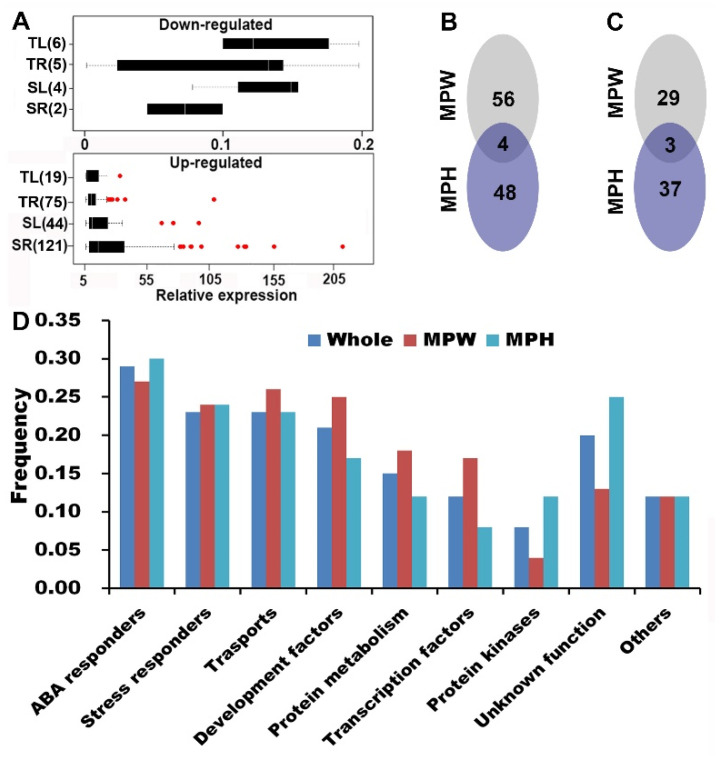
qRT-PCR-verified candidate genes that confer drought tolerance in the CCSP. (**A**) Boxplots, quantitative Real-time PCR (qRT-PCR) was performed after one h PEG-treatment. The Boxplots of 25, 80, 48 and 123 significantly differential-expressed genes (more than five-folds) in the leaf and root of drought tolerance accession (TL, TR) and drought sensitive accession (SL, SR). The red dots in the up-grated graph are outliers with a relative expression value of more than 1.5 times the interquartile range. (**B**) The Venn-Diagram of 108 qRT-PCR-verified genes between MPW-SC-QTL and MPH-SC-QTL. (**C**) The Venn-Diagram of 69 qRT-PCR-verified genes between MPW-LC-QTL and MPH-LC-QTL. (**D**) GO functional classification of the verified drought tolerance candidate genes.

Based on the soybean reference genome of Glyma.Wm82.a1.v1.1 (http://www.soybase.org), a total of 354 candidate genes within or neighboring to the 135 SNPLDBs were annotated for MPW and MPH (Table 7). To verify the candidate genes, qRT-PCR was carried out by using two genotypes from the CCSP, drought tolerant N23644 (T) and drought-sensitive N00710 (S). A total of 177 annotated genes displayed differential expressions at more than five-folds in at least one of the four pairs of comparisons, which were the combinations of leaf (L) and root (R) of N23644 (T) and N00710 (S), i.e., TL, TR, SL and SR categories (Table S4). There showed 6, 5, 4 and 2 down-regulated genes (with expression ranging in 0.11~0.20, 0.02~0.20, 0.09~0.16 and 0.06~0.11, respectively) and 19, 75, 44 and 121 up-regulated genes (with expression ranging in 5.05~32.79, 5.06~108.38, 5.17~96.00 and 5.19~211.57) in TL, TR, SL and SR categories, respectively, with some shared among the categories (Figure 3A, Table S4). In a total, 177 candidate genes were validated, in which, 92 and 92 (with overlapped ones) drought-responsive candidate genes were located in or close to the 52 and 44 (92 in total) SNPLDBs that were associated with MPW and MPH, respectively (Table 7). Among the 177 candidate genes, 69 ones were from 24 LC-major QTL, 108 from 68 SC-major QTL and 7 from 4 shared QTL (Table 7, Figure 3B,C).

Among the 177 candidate genes, there were 30 most likely confident candidate genes that should be particularly studied further, including 22 highly expressed candidate genes and 10 candidate genes with their allele phenotypes significantly different at *p* = 0.05 (Table 8, with two shared). According to the results of qRT-PCR, 1, 6, 4 and 11 (22 in total) supper candidates were identified in TL, TR, SL and SR categories (Figure 3A, Table 8, Table S4), respectively, with relative expression values more than 1.5 times of the inter-quartile range based on boxplot. Among them, the most sensitive gene was *Glyma07g18280*, which expressed similar patterns in the leaf of T and in the leaf and root of S, with relative expression values of 16.62 and 1.46, 75.58 and 211.57, respectively. *Glyma07g18280* belonging to iron/ascorbate family oxidoreductases, was involved in multiple biological processes including jasmonic acid stimulus, oxidation-reduction, response to karrikin and so on. *Glyma02g08115* coding *Pip1* protein, is a drought-induced water channel protein, which was predicted to be responsible for water deprivation, salt stress and ABA stimulus (https://www.ncbi.nlm.nih.gov/nucleotide/U27347.1). *Glyma02g26160*, coding lipoxygenase, and *Glyma11g16750*, in aldehyde dehydrogenase family, were both predicted involving in the biological processes of response to water deprivation (Table 8). As for the 10 candidate genes with their allele phenotypes significantly different at *p* = 0.05 (included in the 177 candidate gens), these should be also the confident candidate genes, in which, *Glyma02g08115* and *Glyma03g01262* were also identified from high expression level of qRT-PCR (Table 8) and *Glyma16g27350* was predicted to be a *Sucrose transport protein* (Table 8), whose homologous genes were required for abiotic stress tolerance in an ABA-dependent pathway in *Arabidopsis thaliana* [31]. However, the above potential major candidate genes-alleles are only a small part of the 177 ones, the others are to be explored further. In addition, among the 177 candidate genes, 45 ones contain SNP(s) in the CCSP, including 25 ones with single SNP and 20 ones with multiple SNPs (Table S5). On the 45 candidate genes each with 2–6 alleles, 117 alleles were recognized totally, where 24 alleles from the 10 genes with different allele phenotypes were significantly associated with DT.

In gene ontology enrichment analysis, all the above 177 predicted candidate genes were grouped into nine categories, i.e., ABA responders (51), stress responders (41), transports (41), development factors (38), protein metabolism (26), transcription factors (21), protein kinases (15), unknown function (35) and others (22) (Figure 3D, Table S6). The proportions of the candidate genes over the nine categories were similar for MPW, MPH and shared ones (Figure 3D and Table S6), which indicated that each indicator included all the nine gene categories or a similar set of functional genes. Furthermore, the genes related to the 58 excluded and 16 emerged QTL-alleles changed from LRS to RCS were located on 37 DT QTL, in which 95 verified candidate genes were included, which indicated that more candidate genes were related to the evolutionarily changed loci. Among the 95 verified candidate genes, 25, 25, 18, 27, 14, 12, 16, 14 and 10 ones were involved in the nine GO groups, where ABA responders, stress responders and development factors were also the major categories (Tables S4 and S6). Thus, the five sets of gene ontology enrichment analysis in Table S6 showed a similar functional classification results, indicating that DT in fact is the resulted performance contributed from a series of functional genes, and that the DT gene network composed of the nine category functions determines the DT performance. As we understand, to know the DT genetic mechanism we have to know the whole picture of the genes, and therefore the whole set of the QTL-alleles, rather than the individual QTL-allele or gene-allele.

## 3. Discussion

### 3.1. The Progress of Present Mapping Results in Comparison to Those in the Literature

In the present study, a large germplasm population of 564 accessions from various eco-regions and provinces with wide variation in DT fitted well in the detection of genome-wide DT QTL-alleles through RTM-GWAS. Comparing with the linkage mapping results of DT in SoyBase (www.soybase.org), there were 40 QTL close to (within 1 Mbp) or in a same region as the 39 QTL detected in the present study (Table S7) among all the reported 134 QTL in six RIL (recombinant inbred line) populations [9,10,23,32]. Among the 40 linkage-mapped QTL, eight ones were included in the seven LC-major QTL (with phenotypic contribution ranging from 1.0% to 2.5%) and 32 ones included in the present SC-major QTL. Therefore, these linkage-mapped QTL are only a small part of the 135 QTL detected from RTM-GWAS in the CCSP. Obviously, the RTM-GWAS for a wide-variation population was much more powerful than the linkage mapping on the six RIL populations for detecting DT QTL.

Among the 135 detected QTL, there were some specific ones worthy for further study (Table S8). For example, Gm06_BLOCK576 and Gm08_BLOCK466 were two shared LC-major QTL with high phenotypic contribution; Gm11_BLOCK241 and Gm13_BLOCK338 were strongly differentiated QTL between LRS and RCS; the 16 emerged new DT alleles in RCS located on nine DT loci. These specific QTL involved with the candidate genes related to all the substantial biological processes, might be the most important QTL with most important candidate genes among the 135 QTL and 177 candidate genes.

In addition to the mapped QTL-alleles, candidate genes-alleles were explored further based on high expression of qRT-PCR and gene-allele/haplotype analysis. In the latter, due to low coverage of SNP in the present study, only 117 alleles on 45 genes were detected from the 177 DT candidate genes (Table S5). Among them, 24 alleles from 10 genes were significantly associated with DT indicators, MPW or MPH, but only two genes shared with those from the high expression of qRT-PCR. It can be expected that more gene-alleles can be identified if the sequencing depth increased. Anyway, the present genes-alleles should be the most likely DT candidate genes, which might be used for gene-cloning and marker-assisted selection.

### 3.2. The Efficiency and Usefulness of Genome-Wide QTL Detection through RTM-GWAS

As described by He et al. [29], the major advantage of the innovative RTM-GWAS procedure was powerful in relatively full detection of the genome-wide QTL-allele system with the total phenotypic contribution (*R*^2^) asymptotic to the overall heritability value through marker pre-selection followed with multi-locus multi-allele stepwise regression. It was especially important for population geneticists and breeders to know the complete set of QTL-allele system. Another basic feature of the RTM-GWAS was that a new type of genomic marker SNPLDB with multiple haplotypes per locus can fit the multiple-allele property of germplasm population with the LD decay distance reduced for a better GWAS efficiency. Lu et al. [22] also indicated that the efficiency and the accumulative contribution to the total variation would be substantially improved when using markers with multiple alleles. In RTM-GWAS, the multiple allele effects could be estimated from the stepwise regression, and therefore the QTL-allele matrix could be established for further population genetic study. The previous GWAS procedures primarily focused on detecting few major QTL with total *R*^2^ only accounting for a small part of the phenotypic variation [27]. In the present study, total 135 QTL were detected with a total *R*^2^ of approximately 50.9% for the indicators, while in some cases, such as seed weight, the *R*^2^ was more than 90% when heritability reached 98% [29]. If a regular GWAS procedure was used, the detected QTL was equivalent to only the LC-major QTL part in this study, with the SC-major QTL part not included. According to the qRT-PCR validation results, 177 DT candidate genes were verified in the present study, while under a regular GWAS procedure, among the 177 candidate DT genes, 69 located in 24 LC-major QTL can be detected but the other 108 located in 68 SC-major QTL will be missed. Correspondingly, 135 − 29 = 106 SC-major QTL, 4 − 1 = 3 shared QTL and 454 − 159 = 295 alleles will be also missed (Table S9). The differences in QTL/gene detection power strongly supported the RTM-GWAS strategy for a relatively thorough genome-wide QTL detection.

Furthermore, there might be more DT QTL/genes to be explored because the collective unmapped part of the phenotypic variation (26.6% and 28.9%) has not been further dissected at present (Table 4). In the detected DT QTL system, the individual QTL *R^2^* ranged from 0.1% to 3.5%, indicating that there was no very large-contribution QTL. This phenomenon was also found in maize through a large-scale drought stress QTL mapping program (over 1000 QTL) [33]. In fact, a complex trait is usually conferred with a large number of QTL, each one contributing a small part because the total contribution is limited to the heritability value.

The present study has demonstrated the potential utilization of the DT QTL-allele matrix obtained from RTM-GWAS procedure. One is to represent the DT genetic structure of the whole population, which may help for comparisons among multiple sub-populations which can serve the population genetic study. The other is to predict the optimal crosses for best recombinants. Another is to annotate and detect the responsive genes from which the target genes may be cloned and the GO enrichment analysis can be made for understanding the gene network involving with the biological processes. As for the utilization of QTL-allele matrix in candidate gene finding and cloning, we have provided a detailed example in DT gene system. However, to understand the genetic system that confers DT in soybeans, further studies should focus on each of the gene categories, each of the component indicators and their intersection points.

### 3.3. Understanding the Super-DT-Trait and Its Genetic Constitution

After the establishment of QTL-allele matrices, we realized that the genetic systems of the two indicators MPW and MPH were quite different even for a same DT trait. However, the DT genetic system should be a uniform QTL-allele set or 135 QTL with 454 alleles in the present study, thus, we put the two indicators together as a super-DT-trait for finding DT gene system with its components, MPW and MPH, as its sub-DT-traits. Interestingly, the different indicators conferred by a similar but different set of candidate genes were involved with a similar set of biological processes, including ABA responders, stress responders, transports, developmental factors, etc.. Therefore, all the 177 candidate genes should be the members of the DT genetic system. However, our two indicators are not necessarily a complete set of the super-DT, so is for the detected QTL-allele system and the candidate gene system. Some additional possible indicators, as well as their QTL-alleles and candidate genes, might be involved, but the present results should be the major parts and the similar set of biological processes might be involved in the super-DT-trait. A number of morphological, physiological and biochemical characters such as root depth, osmotic adjustment, ABA content and others have been identified as DT indicators [34,35], but each reflects only a particular case of DT. Thus, their QTL/genes might have been included in those of the plant growth (including yield as the final growth) indicators and might be a part of the QTL/gene network related to DT. As we suppose, our further work should be on exploring the knowledge of the super-DT-trait gene network, especially the interrelationship among the genes in the network based on identifying the individual DT QTL/genes with RTM-GWAS.

In summary, this study is featured with a relatively thorough identification of QTL-allele/candidate-gene system, using an efficient RTM-GWAS procedure, on two comprehensive indicators (MPW and MPH) instead of a single biological characteristic), in a large sample of the CCSP. The key point of the above results is the establishment of relatively full QTL-allele matrices composed of numerous gene functions jointly conferring DT, therefore, demonstrates the complexity of DT genetic system and potential of CCSP in DT breeding.

## 4. Materials and Methods

### 4.1. Plant Materials

A core sample of cultivated soybeans from a collection containing more than 20,000 accessions that were conserved in the National Center for Soybean Improvement, was used in the present study. The materials were collected from 26 provinces in the six soybean eco-regions in China [36], including 319 landraces and 245 released cultivars. The germplasm sample, covering a wide range, is a representative sample of the Chinese cultivated soybean population and designated as CCSP.

### 4.2. Experiment Design and Drought Tolerance Measurements

The experiment was carried out in pots at Nanjing Agricultural University (32.04°N, 118.63°E) in 2011. According to the method reported by Liu et al. [20], the experiment was arranged in a split plot design with five replications, water treatments in major plot, including water-stress (30 mL of water per pot every day) versus water-non-stress (100 mL of water per pot every day) and 564 accessions in sub-plot, including five drought tolerant and five drought sensitive checks. Each pot (Φ25 × H28 cm) was filled with 7 kg of an 85:15 sand-soil mixture. The experiment was conducted in a greenhouse with day/night air temperatures of (28/22) ± 2 °C and 60% relative humidity. To obtain uniform seedlings, the seeds were germinated and the most uniform seedlings were visually selected and transferred to pots. The plants were thinned to two per pot and then treated with the two water regimes from the seventh day after planting. Two growth-related traits, the plant weight and plant height, were evaluated on the 20th day after treatment to assess the DT. The plant height was measured with a ruler before harvest, and the whole plant including shoot and root was dried to a constant weight at 65 °C for 48 h and then weighed.

The two DT indicators, MPW and MPH, were calculated according to the following formula [20]. *M_ik_* = (*X_ik_*−*X_k_*_S_)*/*(*X_k_*_T_−*X_k_*_S_), where *M_ik_* represents the membership index value of *i*th genotype, *k*th replication, and *X* is the relative plant weight or plant height under water stress to non-stress condition. Where *X_k_*_T_ and *X_k_*_S_ represent the average value of five drought-tolerant and five drought-sensitive checks, respectively, in replication *k*. The lager the *M_ik_* value is, the stronger the drought tolerance is.

### 4.3. Genotyping of CCSP and SNPLDB Assembly

The accessions were sequenced using RAD-seq (restriction-site-associated DNA sequencing) at BGI Tech (Shenzhen, China). The genomic DNAs from fresh leaves were processed using the CTAB protocol [37] and sequenced on an HiSeq2000 instrument (Illumina, San Diego, CA, USA) by multiplexed shotgun genotyping method [38] with DNA fragments of 400~700 bp, generating 1.176 billion paired-end reads of 90-bp (including 6-bp index) read lengths (170.85 Gb of sequence), with the most having an approximately × 3.86 depth and 4.57% coverage. All sequence reads were aligned against the genome of Williams 82 [39] using SOAP2 [40]. The RealSFS [41] was used for population SNP-calling based on the Bayesian estimation of locus frequency. The SNPs of 564 accessions were polymorphic with a rate of missing and heterozygous allele calls ≤ 30% and a minor allele frequency (MAF) ≥ 1%. The FastPHASE software [42] was used for genotyping SNP imputation after heterozygous alleles were turned into missing alleles. The final set of SNPs were used to construct SNPLDBs through an accelerated EM algorithm with Haploview 4.2 software [43]. The LD blocks were defined by a default algorithm with 95% confidence intervals except that the maximum distance and minimum MAF were set to 200 kb and 0.01, respectively [44]. Then the SNPs within a LD block were organized into a SNPLDB marker with haplotypes as its alleles. Each SNP outside the LD blocks was also treated as a SNPLDB with only a single SNP. From these analyses, a total of 108,610 SNPs and 24,694 SNPLDBs were identified in the CCSP.

### 4.4. RTM-GWAS and QTL-Allele Matrix of Drought Tolerance in CCSP

The RTM-GWAS software [29] was used to identify the causal loci of DT on the whole genome. In both stages, the top 10 eigenvectors of the genetic similarity matrix built on SNPLDBs were incorporated as covariates to correct the population structure bias. In the first stage of a single-locus model association analysis, a significance level of *p* = 0.05 was set to pre-select the candidate markers. In the second stage, these candidate SNPLDBs were used in a stepwise regression under a multi-locus multi-allele model with the total QTL genetic contribution controlled within the heritability. The QTL with their allele effects were organized into QTL-allele matrix for the respective traits. Furthermore, each QTL-allele matrix was split into LRS and RCS matrices to show the population evolutionary changes from LRS to RCS. In addition, the frequency distribution on each locus was χ^2^-tested for detecting the differentiation between LR and RC subpopulations.

### 4.5. Optimal Cross Prediction

For each indicator, 158,766 possible crosses were predicted from the 564 accessions. In each cross, 10,000 progenies were simulated for their MPW and MPH values based on their respective QTL-allele matrices. The optimal crosses were predicted according to the simulated progeny distributions. If the 95th percentiles of the predicted progeny values for the two indicators are simultaneously greater than the highest parental values, the cross is considered optimal for super-DT-trait. It is because that the QTL-allele systems of the two indicators are quite different (see Results), and each indicator QTL-allele system is considered only a part of DT, thus the two indicators compose of a super-DT-trait while each indicator is a sub-DT trait.

### 4.6. Identification of the Candidate Gene System of Drought Tolerance

From the identified QTL using RTM-GWAS, the candidate genes related to DT were annotated according to the reference genome of Glyma.Wm82.a1.v1.1 [39]. To validate the annotated candidate genes, the qRT-PCR was carried out using the two genotypes selected from the CCSP, drought tolerant N23644 (T) and drought sensitive N00710 (S), exhibiting contrasting drought stress expressed in MPW (0.750 vs. 0.098) and MPH (0.236 vs. −1.323). The seeds were germinated, and the uniform seedlings were transferred to plastic cups filled with culture medium and then grown under greenhouse conditions (28 °C, 16h/8h photoperiod and 60% relative humidity). When the first trifoliate leaves were unfolded, the RNA samples were extracted from the leaves and roots of at least three uniform plants [45] after a quick drought stress treatment with 10% PEG 6000 (polyethylene glycol 6000) in hydroponics at 0 h and 1 h [46]. Then the RNA specimens were used for qRT-PCR. The relative quantity of gene expression was detected with 2^–ΔΔCT^ method [47], using the *60S* expression as the internal standard [48]. A total of 177 confident candidate genes were identified.

In addition to the criterion of high qRT-PCR expressions, the identified 177 confident candidate genes were further tested for their phenotypic difference among the candidate alleles (obtained and grouped from the RAD-seq data of the 564 accessions). The accessions were grouped for their allele type on each locus and F-tested at *p* = 0.05 for their significant differences among candidate alleles for the corresponding DT indicator, MPW or MPH. The candidate genes (in a total of 10) that showed significant differences among their alleles were recognized as confident candidate genes.

## 5. Conclusions

The previous knowledge on DT QTL was mainly from individual crosses involving only few parental materials, which needs to expand to broad germplasm resources. A sample composed of 564 accessions of the CCSP was studied for the DT QTL-allele system with MPW and MPH as indicators, using the innovative RTM-GWAS procedure. In CCSP, DT as a super-trait composed of MPW and MPH sub-traits was conferred by two different (even more) QTL-allele matrices/systems, each with 75 and 64 QTL with 261 and 207 alleles (2–12 per locus), respectively, in a total of 135 QTL with 468 alleles. From which, 10 top crosses were predicted to show large transgressive breeding potentials, and found that 58 LRS alleles disappeared but 16 new ones emerged in RCS during the evolution from LRS to RCS, and the 177 qRT-PCR-verified candidate genes were grouped into 9 categories as a gene network with ABA and stress responders as major parts. The key point of the present study is the establishment of relatively full QTL-allele matrices which includes plentiful QTL with numerous gene functions jointly conferring DT, therefore, are relevant to breeding for DT and to understanding the DT gene network in CCSP.

## Figures and Tables

**Table 1:** Frequency distribution of the two drought tolerance indicators in the CCSP.

Indicator	Population	The Frequency Distribution										Mean	Min	Max	Range	*h* ^2^
MPW	Midpoint	−0.37	−0.19	−0.01	0.17	0.35	0.53	0.71	0.89	1.07	1.25					
	CCSP	5	10	26	97	155	166	71	24	7	3	0.419	−0.362	1.411	1.773	81.30%
	LRS	2	4	12	49	88	102	43	14	4	1	0.433	−0.362	1.273	1.635	
	RCS	3	6	14	48	67	64	28	10	3	2	0.402	−0.323	1.411	1.734	
MPH	Midpoint	−1.32	−0.97	−0.62	−0.27	0.08	0.43	0.78	1.13	1.48	1.83					
	CCSP	3	6	20	128	242	112	31	11	7	4	0.120	−1.323	1.792	3.115	76.00%
	LRS	2	3	8	67	143	58	21	11	3	3	0.151	−1.323	1.792	3.115	
	RCS	1	3	12	61	99	54	10	0	4	1	0.081	−1.214	1.712	2.926	

MPW and MPH represent the membership index values of relative plant weight and plant height, respectively. CCSP is the Chinese cultivated soybean population; LRS and RCS are the landrace subpopulation and released cultivar subpopulation, respectively. The correlation coefficient of the two indicators was 0.55, significant at *p* = 0.01 level. *h*^2^, heritability value calculated from the ANOVA.

**Table 2:** The QTL conferring drought tolerance in terms of MPW in CCSP.

QTL	SNPLDB	A.N.	−Log_10_*P*	*R^2^* (%)	QTL	SNPLDB	A.N.	−Log_10_*P*	*R^2^* (%)
*MPW1.1*	Gm01_149527	2	11.9	1.0	*MPW10.2*	Gm10_BLOCK96	3	5.1	0.5
*MPW1.2*	Gm01_3646943	2	3.2	0.2	*MPW10.3*	Gm10_BLOCK159	7	23.0	2.5
*MPW2.1*	Gm02_BLOCK81	7	5.9	0.8	*MPW10.4*	Gm10_BLOCK229	8	21.5	2.4
*MPW2.2*	Gm02_14594196	2	11.3	1.0	*MPW10.5*	Gm10_38212261	2	8.1	0.7
*MPW2.3*	Gm02_29143788	2	6.9	0.6	*MPW11.1*	Gm11_BLOCK126	4	4.1	0.4
*MPW2.4*	Gm02_BLOCK579	5	14.4	1.5	*MPW11.2*	Gm11_17784579	2	3.3	0.3
*MPW3.1*	Gm03_326463	2	5.3	0.4	*MPW11.3*	Gm11_BLOCK216	5	6.5	0.7
*MPW3.2*	Gm03_BLOCK11	2	7.2	0.6	*MPW11.4*	Gm11_BLOCK241	3	3.6	0.3
*MPW3.3*	Gm03_993540	2	13.8	1.2	*MPW11.5*	Gm11_26892595	2	8.4	0.7
*MPW3.4*	Gm03_BLOCK283	9	12.5	1.6	*MPW12.1*	Gm12_8508827	2	3.5	0.3
*MPW4.1*	Gm04_10567695	2	9.5	0.8	*MPW12.2*	Gm12_BLOCK254	5	3.3	0.4
*MPW4.2*	Gm04_BLOCK295	5	19.3	2.0	*MPW12.3*	Gm12_BLOCK402	3	4.9	0.5
***MPW5.1***	***Gm05_18374832***	**2**	**3.5**	**0.3**	*MPW12.4*	Gm12_BLOCK429	3	8.4	0.8
*MPW5.2*	Gm05_20554448	2	4.6	0.4	*MPW13.1*	Gm13_2179313	2	2.5	0.2
*MPW5.3*	Gm05_33077723	2	2.6	0.2	*MPW13.2*	Gm13_BLOCK338	6	33.0	3.5
*MPW6.1*	Gm06_BLOCK201	4	14.9	1.5	*MPW13.3*	Gm13_BLOCK486	4	13.4	1.3
*MPW6.2*	Gm06_BLOCK264	2	5.0	0.4	*MPW14.1*	Gm14_39515432	2	4.7	0.4
*MPW6.3*	Gm06_BLOCK401	6	4.1	0.5	*MPW15.1*	Gm15_2915560	2	5.1	0.4
*MPW6.4*	Gm06_BLOCK522	5	7.3	0.8	*MPW15.2*	Gm15_BLOCK93	3	4.0	0.4
***MPW6.5***	***Gm06_BLOCK576***	**3**	**13.3**	**1.3**	*MPW15.3*	Gm15_30923425	2	2.2	0.2
*MPW7.1*	Gm07_3177189	2	8.6	0.7	*MPW15.4*	Gm15_BLOCK409	12	8.2	1.3
*MPW7.2*	Gm07_BLOCK229	6	7.5	0.8	*MPW16.1*	Gm16_2912151	2	2.4	0.2
*MPW7.3*	Gm07_BLOCK302	4	2.8	0.3	*MPW16.2*	Gm16_7534785	2	2.5	0.2
*MPW7.4*	Gm07_BLOCK373	3	3.5	0.3	*MPW16.3*	Gm16_BLOCK365	2	9.2	0.8
*MPW8.1*	Gm08_BLOCK49	3	2.8	0.2	*MPW17.1*	Gm17_BLOCK344	7	3.1	0.4
*MPW8.2*	Gm08_BLOCK71	4	4.7	0.5	*MPW17.2*	Gm17_BLOCK388	3	6.2	0.6
*MPW8.3*	Gm08_11056573	2	2.5	0.2	*MPW18.1*	Gm18_BLOCK736	3	3.7	0.3
*MPW8.4*	Gm08_BLOCK165	2	6.2	0.5	*MPW19.1*	Gm19_45043655	2	3.1	0.2
*MPW8.5*	Gm08_BLOCK209	2	9.1	0.8	*MPW19.2*	Gm19_BLOCK554	5	8.2	0.9
***MPW8.6***	***Gm08_BLOCK466***	**9**	**4.3**	**0.7**	*MPW19.3*	Gm19_46882319	2	3.4	0.3
*MPW8.7*	Gm08_BLOCK527	3	7.7	0.7	*MPW20.1*	Gm20_6329124	2	4.2	0.3
*MPW9.1*	Gm09_963514	2	9.6	0.8	*MPW20.2*	Gm20_BLOCK210	4	9.1	0.9
*MPW9.2*	Gm09_2378279	2	11.4	1.0	*MPW20.3*	Gm20_BLOCK429	2	11.8	1.0
*MPW9.3*	Gm09_BLOCK115	7	7.0	0.9	*MPW20.4*	Gm20_BLOCK468	2	2.3	0.2
*MPW9.4*	Gm09_BLOCK142	4	7.0	0.7	***MPW20.5***	***Gm20_39658098***	**2**	**6.3**	**0.5**
*MPW9.5*	Gm09_35353845	2	8.5	0.7	*MPW20.6*	Gm20_41737971	2	2.3	0.2
*MPW9.6*	Gm09_36608762	2	3.1	0.2	*MPW20.7*	Gm20_BLOCK531	5	5.3	0.6
*MPW10.1*	Gm10_BLOCK71	5	16.8	1.8	Total 75		261		54.7

MPW is one of the two drought tolerance indicators in terms of membership index value of plant weight. QTL: a QTL in italic boldface means the locus is shared between MPW and MPH. SNPLDB: the SNPLDB with only single SNP is designated as, for example, “Gm20_6329124” where Gm20 represents Chromosome 20, and “6,329,124” indicates the locus physical position in bp; while the SNPLDB with multiple SNPs (M.SNPLDB) is designated as, for example, “Gm20_BLOCK210” where Gm20 means Chromosome 20, and BLOCK210 represents the 210th M.SNPLDB on this chromosome. The positions of the SNPLDBs are listed in Table S2. A.N.: number of alleles in a SNPLDB in the CCSP. –Log_10_*^P^*: the probability value of a SNPLDB in RTM-GWAS; since the accumulated heritability of the selected QTL was not over the total heritability, such as 81.3% for MPW, we did not use after-stepwise Bonferroni correction for further QTL selection. *R^2^*: genetic contribution to the phenotypic variation of a QTL. The same is true for the later tables.

**Table 3:** The QTL conferring drought tolerance in terms of MPH in CCSP.

QTL	SNPLDB	A.N.	−Log_10_*P*	*R^2^* (%)	QTL	SNPLDB	A.N.	−Log_10_*P*	*R^2^* (%)
*MPH1.1*	Gm01_BLOCK493	4	8.3	0.9	*MPH9.5*	Gm09_41866356	2	2.7	0.2
*MPH1.2*	Gm01_BLOCK546	3	5.6	0.6	*MPH11.1*	Gm11_BLOCK74	2	2.5	0.2
*MPH2.1*	Gm02_161337	2	4.7	0.4	*MPH11.2*	Gm11_BLOCK135	5	5.0	0.6
*MPH2.2*	Gm02_BLOCK299	5	12.1	1.4	*MPH11.3*	Gm11_27967762	2	3.3	0.3
*MPH2.3*	Gm02_50652770	2	7.0	0.6	*MPH11.4*	Gm11_BLOCK344	3	9.0	0.9
*MPH4.1*	Gm04_BLOCK232	8	5.4	0.8	*MPH12.1*	Gm12_32591630	2	3.9	0.3
*MPH4.2*	Gm04_BLOCK490	7	5.1	0.8	*MPH13.1*	Gm13_10222518	2	9.6	0.9
*MPH4.3*	Gm04_43717074	2	2.2	0.2	*MPH13.2*	Gm13_BLOCK177	8	19.1	2.4
*MPH4.4*	Gm04_47582011	2	6.6	0.6	*MPH13.3*	Gm13_23309035	2	6.4	0.6
*MPH5.1*	Gm05_10333534	2	4.1	0.3	*MPH13.4*	Gm13_28457573	2	2.8	0.2
***MPH5.2***	***Gm05_18374832***	**2**	**4.2**	**0.4**	*MPH13.5*	Gm13_BLOCK396	2	5.8	0.5
*MPH6.1*	Gm06_BLOCK172	3	9.7	1.0	*MPH14.1*	Gm14_3106285	2	3.0	0.2
*MPH6.2*	Gm06_BLOCK208	7	8.2	1.1	*MPH14.2*	Gm14_25589678	2	2.4	0.2
*MPH6.3*	Gm06_34868214	2	3.3	0.3	*MPH14.3*	Gm14_BLOCK408	6	12.3	1.5
*MPH6.4*	Gm06_BLOCK491	12	16.7	2.4	*MPH15.1*	Gm15_BLOCK240	5	16.3	1.9
***MPH6.5***	***Gm06_BLOCK576***	**3**	**13.2**	**1.4**	*MPH15.2*	Gm15_31242502	2	3.1	0.3
*MPH7.1*	Gm07_BLOCK25	4	25.9	2.8	*MPH15.3*	Gm15_BLOCK383	9	22.5	2.9
*MPH7.2*	Gm07_16348924	2	2.9	0.2	*MPH16.1*	Gm16_BLOCK67	2	8.7	0.8
*MPH7.3*	Gm07_BLOCK194	2	9.5	0.9	*MPH16.2*	Gm16_BLOCK395	3	15.4	1.6
*MPH7.4*	Gm07_BLOCK272	2	5.6	0.5	*MPH18.1*	Gm18_4912699	2	2.5	0.2
*MPH7.5*	Gm07_30175006	2	2.4	0.2	*MPH18.2*	Gm18_7175261	2	2.4	0.2
*MPH7.6*	Gm07_32919498	2	5.4	0.5	*MPH18.3*	Gm18_BLOCK129	3	7.4	0.8
*MPH7.7*	Gm07_42499533	2	5.6	0.5	*MPH18.4*	Gm18_26517331	2	4.0	0.3
*MPH8.1*	Gm08_BLOCK106	6	15.5	1.9	*MPH18.5*	Gm18_BLOCK434	3	4.0	0.4
*MPH8.2*	Gm08_BLOCK250	3	3.1	0.3	*MPH18.6*	Gm18_BLOCK727	3	3.4	0.4
*MPH8.3*	Gm08_28738663	2	7.8	0.7	*MPH18.7*	Gm18_BLOCK729	3	2.2	0.2
*MPH8.4*	Gm08_30916483	2	6.8	0.6	*MPH19.1*	Gm19_37391411	2	2.4	0.2
***MPH8.5***	***Gm08_BLOCK466***	**9**	**9.1**	**1.3**	*MPH19.2*	Gm19_41440516	2	5.3	0.5
*MPH9.1*	Gm09_4538598	2	2.3	0.2	*MPH19.3*	Gm19_42142070	2	8.5	0.8
*MPH9.2*	Gm09_14134401	2	3.8	0.3	*MPH19.4*	Gm19_42756453	2	4.8	0.4
*MPH9.3*	Gm09_20590481	2	2.8	0.2	*MPH20.1*	Gm20_BLOCK388	4	8.8	1.0
*MPH9.4*	Gm09_37359880	2	3.3	0.3	***MPH20.2***	***Gm20_39658098***	**2**	**6.3**	**0.6**
					Total 64		207		47.1

MPH is the one of the two drought tolerance indicators in terms of membership index value of relative plant height. Please see the notes in Table 2 for other items.

**Table 4:** The detected QTL-allele system that confers drought tolerance in the CCSP.

QTL System	MPW	MPH	Total
**QTL**			
Whole	54.7 (75, 0.2~3.5, 67.0)	47.1 (64, 0.2~2.9, 62.0)	135
LC-major QTL	25.9 (16, 1.0~3.5, 47.3)	24.6 (14, 1.0~2.9, 52.2)	29
SC-major QTL	28.8 (59, 0.2~0.9, 52.7)	22.5 (50, 0.2~ 0.9, 47.8)	106
Unmapped minor QTL	26.6	28.9	
Total contribution (*h*^2^)	81.3	76.0	
Shared QTL/marker	2.8 (4, 0.3~1.3, 3.4)	3.7 (4, 0.4~1.4, 4.9)	4
**Allele**			
Whole	261 (3.4, 2~12)	207 (3.2, 2~12)	452
Shared allele	16	16	16
Positive allele	127 (0.003~0.817)	106 (0.001~1.125)	229
Negative allele	134 (−0.668~0.001)	101 (−1.516~−0.013)	230

MPW and MPH represent the membership index values of relative plant weight and plant height, respectively. In the QTL system column, “Whole” is the total QTL; LC-major QTL, large-contribution major QTL with genetic contribution (*R^2^*) of more than 1.0%; SC-major QTL, small-contribution major QTL with *R^2^* less than 1.0%; and Shared QTL/marker, a QTL/marker shared with the two indicators. In the columns of the two indicators for “QTL”, the number outside the parentheses is the total *R^2^* of the corresponding QTL, the first in parentheses is the number of QTL, the second is a range of *R^2^* for the individual QTL and the third is the portion of QTL contribution to *h*^2^ (the total genetic contribution). In the columns of the two indicators for “Allele”, the number outside the parentheses is the total alleles, the first number in parentheses for “Whole” is the average number of alleles per locus followed by a range of allele numbers per locus, and the number in parentheses for “Positive allele” and “Negative allele” are ranges of corresponding allele effects. The *R^2^* of the unmapped minor QTL is calculated from the total contribution (*h^2^*) ‒ the contribution of whole detected QTL. In the total column, the number is the total detected number of QTL/markers or alleles with the duplicated ones not in the counts.

**Table 5:** Alleles changed from landrace subpopulation to released cultivar subpopulation.

QTL	Trait	a1	a2	a3	a4	a5	a6	a7	a8	a9	a10	a11	a12
Gm01_BLOCK493	MPH				N								
Gm02_BLOCK299	MPH	X		X									
Gm02_BLOCK579	MPW	X	X										
Gm03_BLOCK283	MPW	X	X										
Gm04_BLOCK232	MPH	N	X						X				
Gm04_BLOCK490	MPH	N											
Gm06_BLOCK208	MPH	X	X					X					
Gm06_BLOCK491	MPH		X			X	X	X				X	
Gm06_BLOCK522	MPW					N							
***Gm06_BLOCK576***	***MPW MPH***			N									
Gm07_BLOCK229	MPW	X					X						
Gm07_BLOCK302	MPW	N		N									
Gm08_BLOCK71	MPW	X	X		X								
Gm08_BLOCK106	MPH						X						
Gm08_11056573	MPW	X											
Gm08_BLOCK250	MPH			X									
***Gm08_BLOCK466***	***MPW MPH***	X	X										
Gm08_BLOCK49	MPW	X											
Gm08_BLOCK527	MPW			X									
Gm09_BLOCK115	MPW							X					
Gm09_BLOCK142	MPW				N								
Gm10_BLOCK96	MPW	X											
Gm10_BLOCK159	MPW	X											
Gm10_BLOCK229	MPW						X	X	X				
Gm11_BLOCK135	MPH					X							
Gm11_BLOCK216	MPW				N	X							
Gm12_BLOCK254	MPW	X		X									
Gm13_BLOCK177	MPH								X				
Gm13_BLOCK486	MPW		X										
Gm14_BLOCK408	MPH	X	X				X						
Gm15_BLOCK93	MPW			X									
Gm15_BLOCK240	MPH		X										
Gm15_30923425	MPW	N											
Gm15_BLOCK383	MPH					X			X				
Gm15_BLOCK409	MPW	X			N			N		N			
Gm17_BLOCK344	MPW	X	X	X				X					
Gm18_BLOCK727	MPH	X											
Gm19_37391411	MPH	X											
Gm19_42142070	MPH	N											
Gm19_BLOCK554	MPW	N				X							
Gm20_BLOCK210	MPW	X		X									
***Gm20_39658098***	***MPW MPH***	N											
Gm20_BLOCK531	MPW	X											

a1–a12 are the alleles of each QTL, arranged in a rising order according to their effect value. The cells marked with white (negative effect) and gray (positive effect) are all alleles in CCSP. The cells with X are alleles excluded in released cultivar subpopulation. The cells with N are alleles newly emerged in released cultivar subpopulation (but not existed in landrace subpopulation). The QTL shared by MPW and MPH is in italic boldface.

**Table 6:** The predicted optimal crosses for drought tolerance according to the 95th percentile values of progenies.

Code	Cross		Eco-region	MPW	MPH	WAV
1	P1	N25340 *	II	1.223 (35, 40)	1.439 (35, 29)	1.327
	P2	N25258 *	I	1.411 (41, 34)	1.712 (37, 27)	1.556
	Progeny (95th percentile)			2.392	2.552	2.469
2	P1	N25340 *	II	1.223 (35, 40)	1.439 (35, 29)	1.327
	P2	N23640	IV	1.273 (36, 39)	0.808 (37, 27)	1.048
	Progeny (95th percentile)			2.385	2.664	2.520
3	P1	N07686	IV	1.131 (34,41)	0.561 (36, 28)	0.856
	P2	N25340 *	II	1.223 (35, 40)	1.439 (35, 29)	1.327
	Progeny (95th percentile)			2.330	2.762	2.539
4	P1	N25340 *	II	1.223 (35, 40)	1.439 (35, 29)	1.327
	P2	N21175 *	III	1.071 (36, 22)	1.168 (35, 29)	1.118
	Progeny (95th percentile)			2.194	2.715	2.446
5	P1	N25340 *	II	1.223 (35, 40)	1.439 (35, 29)	1.327
	P2	N04650 *	IV	0.988 (36, 39)	0.110 (34, 30)	0.564
	Progeny (95th percentile)			2.164	2.244	2.203
6	P1	N25148	II	0.708 (35, 39)	0.084 (33, 31)	0.407
	P2	N25340 *	II	1.223 (35, 40)	1.439 (35, 29)	1.327
	Progeny (95th percentile)			2.146	2.339	2.239
7	P1	N25340 *	II	1.223 (35, 40)	1.439 (35, 29)	1.327
	P2	N25321	I	0.645 (40, 35)	0.059 (36, 28)	0.362
	Progeny (95th percentile)			2.143	2.347	2.242
8	P1	N24359	VI	0.716 (34, 41)	0.477 (35, 29)	0.601
	P2	N25340 *	II	1.223 (35, 40)	1.439 (35, 29)	1.327
	Progeny (95th percentile)			2.140	2.417	2.274
9	P1	N25340 *	II	1.223 (35, 40)	1.439 (35, 29)	1.327
	P2	N24595	I	0.818 (37, 38)	0.321 (33, 31)	0.578
	Progeny (95th percentile)			2.126	2.272	2.197
10	P1	N25340 *	II	1.223 (35, 40)	1.439 (35, 29)	1.327
	P2	N24614 *	III	1.005 (36, 39)	1.668 (37, 27)	1.325
	Progeny (95th percentile)			2.107	3.135	2.604

In the column of cross, * represents the accessions made among the top 12 selections with high drought tolerance; the predicted 95th percentile is obtained from a simulation with 10, 000 progenies per cross. Eco-region: I: Northern single cropping, spring planting eco-region; II: Huanghuaihai double cropping, spring and summer planting eco-region; III: Middle and lower Changjiang valley double cropping, spring and summer planting eco-region; IV: Central south multiple cropping, spring, summer and autumn planting eco-region; and VI: South China tropical multiple cropping, all-season planting eco-region. In the columns of the two indicators, the numbers in parentheses are the numbers of positive and negative alleles in a parent. In the WAV column is the weighted average value of membership indices with their heritability values as the weights.

**Table 7:** The annotated and verified gene systems that confer drought tolerance in the CCSP.

Gene System	MPW	MPH	Total
Annotated genes in detected QTL	193 (75)	181 (64)	354 (135)
qRT-PCR-verified genes	92 (52)	92 (44)	177 (92)
Verified genes in LC-major QTL	30 (13)	40 (12)	69 (24)
Verified genes in SC-major QTL	62 (39)	52 (32)	108 (68)
Verified genes in shared QTL	7 (4)	7 (4)	7 (4)

A total of 354 genes were annotated within or neighboring the detected SNPLDBs. Their relative expressions were analyzed using qRT-PCR under PEG treatment vs. non-treatment conditions, from which 177 candidate genes were verified for the two indicators. The number in parentheses is the number of detected QTL hosting the candidate genes.

**Table 8:** The 30 most likely candidate DT genes identified from high expressions in qRT-PCR or significant difference among candidate gene-alleles in ANOVA (two ones identified from both criteria).

	Gene	SNPLDB	Trait	Putative Function
	The 22 candidate genes with high expression under PEG condition			
1	*Glyma05g16373*	Gm05_18374832	MPW, MPH	Ubiquitin Carboxyl-Terminal Hydrolase
**2**	***Glyma03g01262***	**Gm03_993540**	**MPW**	**Kelch Motif; F-Box Domain**
**3**	***Glyma13g40260***	**Gm13_BLOCK486**	**MPW**	**SNARE Domain**
4	*Glyma02g08115*	Gm02_BLOCK81	MPW	Major Intrinsic Protein
5	*Glyma02g08130*	Gm02_BLOCK81	MPW	Transferase Family
6	*Glyma05g17470*	Gm05_20554448	MPW	Leucine Rich Repeat; NB-ARC Domain
7	*Glyma14g32430*	Gm14_39515432	MPW	Protein Phosphatase 2C
8	*Glyma15g13420*	Gm15_BLOCK93	MPW	Unknown Function
9	*Glyma15g13430*	Gm15_BLOCK93	MPW	Pyridoxal-Dependent Decarboxylase Conserved Domain
10	***Glyma02g26160***	**Gm02_BLOCK299**	**MPH**	**PLAT/LH2 Domain**
11	***Glyma06g19220***	**Gm06_BLOCK172**	**MPH**	**Unknown Function**
12	***Glyma06g21570***	**Gm06_BLOCK208**	**MPH**	**Unknown Function**
13	***Glyma06g21584***	**Gm06_BLOCK208**	**MPH**	**Metalloprotease**
14	***Glyma06g40170***	**Gm06_BLOCK491**	**MPH**	**Protein Tyrosine Kinase**
15	***Glyma14g26830***	**Gm14_BLOCK408**	**MPH**	**Unknown Function**
16	***Glyma14g26960***	**Gm14_BLOCK408**	**MPH**	**Protein Tyrosine Kinase**
17	*Glyma04g33540*	Gm04_BLOCK490	MPH	Unknown Function
18	*Glyma07g16651*	Gm07_16348924	MPH	Unknown Function
19	*Glyma07g18280*	Gm07_BLOCK194	MPH	2OG-Fe(II) OxygenaseSuperfamily
20	*Glyma11g16750*	Gm11_BLOCK135	MPH	Aldehyde Dehydrogenase Family
21	*Glyma16g06770*	Gm16_BLOCK67	MPH	Ankyrin Repeat
22	*Glyma18g52470*	Gm18_BLOCK729	MPH	Unknown Function
	The ten genes with significantly different allele phenotypes at *p* = 0.05			
4	*Glyma02g08115*	Gm02_BLOCK81	MPW	Major Intrinsic Protein
**2**	***Glyma03g01262***	**Gm03_993540**	**MPW**	**Kelch Motif; F-Box Domain**
23	*Glyma10g29340*	Gm10_38212261	MPW	Rangap1-Interacting Protein-Related
24	*Glyma12g32950*	Gm12_BLOCK429	MPW	Unknown Function
25	*Glyma16g27350*	Gm16_BLOCK365	MPW	Sugar Transporter
26	***Glyma06g40240***	**Gm06_BLOCK491**	**MPH**	**S-Locus Glycoprotein Family**
27	***Glyma07g03770***	**Gm07_BLOCK25**	**MPH**	**Unknown Function**
28	*Glyma07g37440*	Gm07_42499533	MPH	Glycosyl Hydrolases Family 28
29	***Glyma08g14200***	**Gm08_BLOCK106**	**MPH**	**PPR Repeat**
30	***Glyma16g32180***	**Gm16_BLOCK395**	**MPH**	**Putative Methyltransferase**

The candidate gene and corresponding QTL in boldface represent the QTL is a large-contribution major QTL. The ten genes with allele/haplotype phenotypes significantly different at *p* = 0.05 are also chosen from the 177 candidate genes through F-test for their significant different allele phenotypes among the 564 accessions. See the text for details.

## Supplementary Materials

Supplementary Materials can be found at https://www.mdpi.com/1422-0067/21/14/4830/s1.

## Abbreviations

DT	Drought tolerance
MPW	Membership index value of relative plant weight
MPH	Membership index value of relative plant height
RTM-GWAS	Restricted two-stage multi-locus multi-allele genome-wide association study
qRT-PCR	Quantitative Real-time PCR
CCSP	Chinese cultivated soybean population
QTL	Quantitative trait locus
SNPLDB	SNP linkage disequilibrium block
