# Supplementary material for: Comprehensive Identification of Drought Tolerance QTL-Allele and Candidate Gene Systems in Chinese Cultivated Soybean Population
Source: Int J Mol Sci. 2020 Jul 8;21(14):4830. doi: 10.3390/ijms21144830 (PMC7402128; doi:10.3390/ijms21144830)
Supplement: Supplementary file 1 [file ijms-21-04830-s001.pdf]

Supplementary Tables

Table S1. Highly drought-tolerant and highly drought-sensitive accessions in the CCSP.

Code	Province	Eco-Region	Variety Type	MPW	MPH
		Highly drought-tolerant accession			
N25258	Jilin	I	RC	1.411	1.712
N25340	Shandong	II	RC	1.223	1.439
N09580	Shandong	II	RC	0.933	1.568
N08276	Jiangxi	IV	LR	0.849	1.576
N21043	Jiangxi	IV	LR	0.653	1.656
N21222	Anhui	II	LR	0.545	1.792
N08814	Liaoning	I	RC	0.673	1.513
**N21175**	**Jiangsu**	**III**	**LR**	**1.071**	**1.168**
**N24614**	**Hunan**	**III**	**LR**	**1.005**	**1.668**
**N04650**	**Sichuan**	**IV**	**LR**	**0.988**	**0.110**
**N02549**	**Guizhou**	**V**	**LR**	**0.971**	**1.546**
**N23583**	**Gansu**	**II**	**LR**	**0.965**	**0.508**
		Highly drought-sensitive accession			
N23716	Beijing	II	RC	-0.033	-1.214
N00710	Fujian	IV	LR	0.098	-1.323
N25174	Henan	II	RC	-0.201	-0.995
N24280	Liaoning	I	LR	-0.163	-0.925
N04436	Jiangxi	IV	LR	0.135	-1.240
N25261	Jilin	I	RC	-0.205	-0.870
N05433	Guangdong	VI	LR	-0.084	-0.907
**N25245**	**Jilin**	**I**	**RC**	**-0.026**	**-0.234**
**N02840**	**Shanxi**	**II**	**LR**	**0.000**	**-0.556**
**N25415**	**Hunan**	**IV**	**RC**	**0.002**	**0.133**
**N05410**	**Guangxi**	**VI**	**LR**	**0.008**	**-0.258**
**N23544**	**Jilin**	**I**	**LR**	**0.016**	**0.915**

CCSP is the Chinese cultivated soybean germplasm population; LRS and RCS are the landrace subpopulation and released cultivar subpopulation, respectively. The five highly drought tolerant accessions and five highly drought sensitive ones used as checks to calculate membership index value are in boldface. Eco-region: I: Northern single cropping, spring planting eco-region; II: Huanghuaihai double cropping, spring and summer planting eco-region; III: Middle and lower Changjiang valley double cropping, spring and summer planting eco-region; IV: Central south multiple cropping, spring, summer and autumn planting eco-region; and VI: South China tropical multiple cropping, all-season planting eco-region. MPW and MPH represent the membership indices of plant weight and plant height, respectively.

Table S2. The positions of QTL conferring drought tolerance in CCSP.

QTL	SNPLDB	Position	QTL	SNPLDB	Position
MPW			MPH		
MPW1.1	Gm01_149527	149527	MPH1.1	Gm01_BLOCK493	47264933_47293823
MPW1.2	Gm01_3646943	3646943	MPH1.2	Gm01_BLOCK546	51109836_51114441
MPW2.1	Gm02_BLOCK81	6273618_6448949	MPH2.1	Gm02_161337	161337
MPW2.2	Gm02_14594196	14594196	MPH2.2	Gm02_BLOCK299	26925431_26940852
MPW2.3	Gm02_29143788	29143788	MPH2.3	Gm02_50652770	50652770
MPW2.4	Gm02_BLOCK579	50037068_50084304	MPH4.1	Gm04_BLOCK232	17899360_18006649
MPW3.1	Gm03_326463	326463	MPH4.2	Gm04_BLOCK490	39059520_39224224
MPW3.2	Gm03_BLOCK11	682776_682932	MPH4.3	Gm04_43717074	43717074
MPW3.3	Gm03_993540	993540	MPH4.4	Gm04_47582011	47582011
MPW3.4	Gm03_BLOCK283	27441939_27532289	MPH5.1	Gm05_10333534	10333534
MPW4.1	Gm04_10567695	10567695	MPH5.2	Gm05_18374832	18374832
MPW4.2	Gm04_BLOCK295	23842345_23898799	MPH6.1	Gm06_BLOCK172	15431276_15435935
MPW5.1	Gm05_18374832	18374832	MPH6.2	Gm06_BLOCK208	18122798_18288672
MPW5.2	Gm05_20554448	20554448	MPH6.3	Gm06_34868214	34868214
MPW5.3	Gm05_33077723	33077723	MPH6.4	Gm06_BLOCK491	43295770_43445956
MPW6.1	Gm06_BLOCK201	17499686_17547717	MPH6.5	Gm06_BLOCK576	49809899_49810066
MPW6.2	Gm06_BLOCK264	22103834_22103856	MPH7.1	Gm07_BLOCK25	2596793_2621710
MPW6.3	Gm06_BLOCK401	37416804_37534374	MPH7.2	Gm07_16348924	16348924
MPW6.4	Gm06_BLOCK522	45336128_45364730	MPH7.3	Gm07_BLOCK194	18099542_18115758
MPW6.5	Gm06_BLOCK576	49809899_49810066	MPH7.4	Gm07_BLOCK272	26276821_26276947
MPW7.1	Gm07_3177189	3177189	MPH7.5	Gm07_30175006	30175006
MPW7.2	Gm07_BLOCK229	21443464_21515277	MPH7.6	Gm07_32919498	32919498
MPW7.3	Gm07_BLOCK302	30203444_30235313	MPH7.7	Gm07_42499533	42499533
MPW7.4	Gm07_BLOCK373	36845443_36846591	MPH8.1	Gm08_BLOCK106	10280416_10330725
MPW8.1	Gm08_BLOCK49	4919887_4920152	MPH8.2	Gm08_BLOCK250	22171249_22184601
MPW8.2	Gm08_BLOCK71	7235499_7260878	MPH8.3	Gm08_28738663	28738663
MPW8.3	Gm08_11056573	11056573	MPH8.4	Gm08_30916483	30916483
MPW8.4	Gm08_BLOCK165	16183060_16183086	MPH8.5	Gm08_BLOCK466	40018947_40100361
MPW8.5	Gm08_BLOCK209	18538004_18538077	MPH9.1	Gm09_4538598	4538598
MPW8.6	Gm08_BLOCK466	40018947_40100361	MPH9.2	Gm09_14134401	14134401
MPW8.7	Gm08_BLOCK527	44688033_44689115	MPH9.3	Gm09_20590481	20590481
MPW9.1	Gm09_963514	963514	MPH9.4	Gm09_37359880	37359880
MPW9.2	Gm09_2378279	2378279	MPH9.5	Gm09_41866356	41866356
MPW9.3	Gm09_BLOCK115	8621107_8819963	MPH11.1	Gm11_BLOCK74	6486267_6486274
MPW9.4	Gm09_BLOCK142	11337674_11393438	MPH11.2	Gm11_BLOCK135	12344649_12534077
MPW9.5	Gm09_35353845	35353845	MPH11.3	Gm11_27967762	27967762
MPW9.6	Gm09_36608762	36608762	MPH11.4	Gm11_BLOCK344	30564343_30628745
MPW10.1	Gm10_BLOCK71	5491148_5560788	MPH12.1	Gm12_32591630	32591630
MPW10.2	Gm10_BLOCK96	7026382_7036759	MPH13.1	Gm13_10222518	10222518
MPW10.3	Gm10_BLOCK159	12053290_12110973	MPH13.2	Gm13_BLOCK177	12726894_12922852
MPW10.4	Gm10_BLOCK229	20348469_20509845	MPH13.3	Gm13_23309035	23309035
MPW10.5	Gm10_38212261	38212261	MPH13.4	Gm13_28457573	28457573
MPW11.1	Gm11_BLOCK126	11286334_11300481	MPH13.5	Gm13_BLOCK396	32853739_32853960
MPW11.2	Gm11_17784579	17784579	MPH14.1	Gm14_3106285	3106285
MPW11.3	Gm11_BLOCK216	19260997_19456210	MPH14.2	Gm14_25589678	25589678
MPW11.4	Gm11_BLOCK241	21939990_22028918	MPH14.3	Gm14_BLOCK408	32922809_33122741
MPW11.5	Gm11_26892595	26892595	MPH15.1	Gm15_BLOCK240	20265788_20347922
MPW12.1	Gm12_8508827	8508827	MPH15.2	Gm15_31242502	31242502
MPW12.2	Gm12_BLOCK254	21639072_21792597	MPH15.3	Gm15_BLOCK383	33379981_33579667
MPW12.3	Gm12_BLOCK402	34418573_34433816	MPH16.1	Gm16_BLOCK67	6115682_6115697
MPW12.4	Gm12_BLOCK429	36325875_36326233	MPH16.2	Gm16_BLOCK395	35357235_35358822
MPW13.1	Gm13_2179313	2179313	MPH18.1	Gm18_4912699	4912699
MPW13.2	Gm13_BLOCK338	28533415_28666500	MPH18.2	Gm18_7175261	7175261
MPW13.3	Gm13_BLOCK486	40737493_40748440	MPH18.3	Gm18_BLOCK129	9691839_9691905
MPW14.1	Gm14_39515432	39515432	MPH18.4	Gm18_26517331	26517331

QTL	SNPLDB	Position	QTL	SNPLDB	Position
MPW15.1	Gm15_2915560	2915560	MPH18.5	Gm18_BLOCK434	39409732_39410305
MPW15.2	Gm15_BLOCK93	10005107_10092800	MPH18.6	Gm18_BLOCK727	60984735_60985531
MPW15.3	Gm15_30923425	30923425	MPH18.7	Gm18_BLOCK729	61046143_61063972
MPW15.4	Gm15_BLOCK409	36255072_36454998	MPH19.1	Gm19_37391411	37391411
MPW16.1	Gm16_2912151	2912151	MPH19.2	Gm19_41440516	41440516
MPW16.2	Gm16_7534785	7534785	MPH19.3	Gm19_42142070	42142070
MPW16.3	Gm16_BLOCK365	31363301_31363322	MPH19.4	Gm19_42756453	42756453
MPW17.1	Gm17_BLOCK344	32699950_32806483	MPH20.1	Gm20_BLOCK388	33593168_33630697
MPW17.2	Gm17_BLOCK388	35788429_35788495	MPH20.2	Gm20_39658098	39658098
MPW18.1	Gm18_BLOCK736	61478767_61495577	Total	64	
MPW19.1	Gm19_45043655	45043655			
MPW19.2	Gm19_BLOCK554	45721471_45729322			
MPW19.3	Gm19_46882319	46882319			
MPW20.1	Gm20_6329124	6329124			
MPW20.2	Gm20_BLOCK210	17219843_17346621			
MPW20.3	Gm20_BLOCK429	36186946_36187233			
MPW20.4	Gm20_BLOCK468	38753573_38753583			
MPW20.5	Gm20_39658098	39658098			
MPW20.6	Gm20_41737971	41737971			
MPW20.7	Gm20_BLOCK531	44594152_44673992			
Total	75				

MPW and MPH represent the membership index values of relative plant weight and plant height, respectively. SNPLDB, SNP linkage disequilibrium block. Position, the physical position of SNPLDB according to the reference genome of Glyma.Wm82.a1.v1.1.

Table S3. The allele frequency of the landrace subpopulation (LRS) and the released cultivar subpopulation (RCS) on the 87 differentiated loci.

SNPLDB	Trait	Allele	Effect	CCSP	LRS	RCS	SNPLDB	Trait	Allele	Effect	CCSP	LRS	RCS
Gm01_BLOCK493	MPH	a1	-0.384	0.885	0.912	0.849	Gm10_BLOCK159	MPW	a1	0.076	0.580	0.693	0.433
		a2	-0.360	0.067	0.022	0.127			a2	0.055	0.230	0.154	0.331
		a3	-0.360	0.046	0.066	0.020			a3	0.105	0.067	0.060	0.078
		a4	1.103	0.002	0.000	0.004			a4	0.111	0.066	0.041	0.098
Gm01_BLOCK546	MPH	a1	0.116	0.849	0.850	0.849			a5	-0.049	0.050	0.047	0.053
		a3	0.142	0.131	0.119	0.147			a6	-0.009	0.005	0.003	0.008
		a2	-0.258	0.020	0.031	0.004			a7	-0.290	0.002	0.003	0.000
Gm02_BLOCK81	MPW	a1	0.021	0.505	0.549	0.449	Gm10_BLOCK229	MPW	a1	-0.129	0.621	0.734	0.473
		a2	0.037	0.310	0.317	0.302			a2	-0.604	0.129	0.094	0.176
		a3	-0.013	0.089	0.078	0.102			a3	-0.069	0.115	0.113	0.118
		a4	-0.025	0.046	0.006	0.098			a4	-0.074	0.087	0.031	0.159
		a5	0.164	0.028	0.019	0.041			a5	-0.197	0.035	0.006	0.073
		a6	0.045	0.018	0.028	0.004			a6	0.365	0.009	0.016	0.000
		a7	-0.229	0.004	0.003	0.004			a7	0.005	0.002	0.003	0.000
Gm02_14594196	MPW	G	-0.082	0.959	0.931	0.996			a8	0.712	0.002	0.003	0.000
		A	0.082	0.041	0.069	0.004	Gm10_38212261	MPW	G	0.038	0.855	0.824	0.894
Gm02_BLOCK299	MPH	a1	-0.060	0.810	0.793	0.833			A	-0.038	0.145	0.176	0.106
		a2	0.485	0.129	0.103	0.163	Gm11_BLOCK126	MPW	a1	-0.041	0.392	0.323	0.482
		a3	0.118	0.032	0.053	0.004			a2	-0.035	0.365	0.505	0.184
		a4	0.098	0.023	0.041	0.000			a3	-0.068	0.225	0.147	0.327
		a5	-0.641	0.005	0.009	0.000			a4	0.743	0.018	0.025	0.008
Gm02_BLOCK579	MPW	a1	0.158	0.745	0.715	0.784	Gm11_BLOCK135	MPH	a1	-0.287	0.569	0.536	0.612
		a2	0.146	0.144	0.135	0.155			a2	-0.205	0.367	0.364	0.371
		a3	0.176	0.096	0.122	0.061			a3	-0.204	0.037	0.056	0.012
		a4	-0.100	0.012	0.022	0.000			a4	0.058	0.025	0.041	0.004
		a5	-0.379	0.004	0.006	0.000			a5	0.639	0.002	0.003	0.000
Gm03_993540	MPW	C	0.079	0.883	0.831	0.951	Gm11_BLOCK216	MPW	a1	-0.206	0.716	0.596	0.873
		A	-0.079	0.117	0.169	0.049			a2	-0.272	0.188	0.260	0.094
Gm03_BLOCK283	MPW	a1	0.052	0.360	0.348	0.376			a3	-0.213	0.092	0.141	0.029
		a2	0.058	0.300	0.270	0.339			a4	0.735	0.002	0.003	0.000
		a3	0.110	0.129	0.119	0.143			a5	-0.044	0.002	0.000	0.004
		a4	0.090	0.067	0.069	0.065	Gm11_BLOCK241	MPW	a1	0.015	0.649	0.492	0.853
		a5	0.024	0.060	0.097	0.012			a2	0.054	0.278	0.398	0.122
		a6	0.071	0.044	0.031	0.061			a3	-0.069	0.073	0.110	0.024
		a7	0.004	0.028	0.050	0.000	Gm11_27967762	MPH	C	-0.087	0.949	0.931	0.971
		a8	0.171	0.007	0.013	0.004			T	0.087	0.051	0.069	0.029
		a9	-0.579	0.004	0.003	0.000	Gm11_BLOCK344	MPH	a1	0.021	0.883	0.818	0.967
Gm04_10567695	MPW	C	-0.173	0.980	0.994	0.963			a2	-0.117	0.074	0.113	0.024
		T	0.173	0.020	0.006	0.037			a3	0.096	0.043	0.069	0.008
Gm04_BLOCK232	MPH	a1	0.145	0.741	0.668	0.837	Gm12_8508827	MPW	T	-0.026	0.715	0.790	0.616
		a2	0.080	0.142	0.191	0.078			G	0.026	0.285	0.210	0.384
		a3	0.286	0.066	0.094	0.029	Gm12_BLOCK254	MPW	a1	0.059	0.888	0.915	0.853
		a4	0.379	0.025	0.034	0.012			a2	0.147	0.043	0.016	0.078
		a5	0.005	0.021	0.006	0.041			a3	-0.053	0.041	0.019	0.069
		a6	-1.516	0.002	0.000	0.004			a4	0.014	0.023	0.041	0.000
		a7	0.812	0.002	0.003	0.000			a5	-0.167	0.005	0.009	0.000
		a8	-0.191	0.002	0.003	0.000	Gm12_BLOCK402	MPW	a1	0.019	0.844	0.887	0.788
Gm04_BLOCK295	MPW	a1	0.054	0.759	0.683	0.857			a2	0.077	0.115	0.100	0.135
		a2	-0.022	0.094	0.132	0.045			a3	-0.096	0.041	0.013	0.078
		a3	-0.064	0.083	0.097	0.065	Gm12_BLOCK429	MPW	a1	-0.070	0.566	0.489	0.665
		a4	-0.123	0.043	0.056	0.024			a2	-0.011	0.284	0.339	0.212
		a5	0.154	0.021	0.031	0.008			a3	0.081	0.151	0.172	0.122
Gm04_BLOCK490	MPH	a1	0.071	0.433	0.464	0.392	Gm13_BLOCK177	MPH	a1	-0.059	0.456	0.530	0.359
		a2	0.115	0.199	0.235	0.151			a2	-0.139	0.183	0.160	0.212
		a3	0.009	0.145	0.088	0.220			a3	-0.891	0.126	0.100	0.159
		a4	-0.172	0.140	0.100	0.192			a4	-0.021	0.085	0.013	0.180
		a5	0.142	0.069	0.100	0.029			a5	0.075	0.074	0.078	0.069
		a6	0.027	0.009	0.013	0.004			a6	-0.201	0.062	0.100	0.012
		a7	-0.191	0.005	0.000	0.012			a7	0.298	0.011	0.013	0.008
Gm04_47582011	MPH	T	0.203	0.970	0.953	0.992			a8	0.938	0.004	0.006	0.000
		G	-0.203	0.030	0.047	0.008	Gm13_28457573	MPH	T	-0.103	0.963	0.940	0.992
Gm05_33077723	MPW	C	-0.065	0.949	0.991	0.894			G	0.103	0.037	0.060	0.008

SNPLDB	Trait	Allele	Effect	CCSP	LRS	RCS	SNPLDB	Trait	Allele	Effect	CCSP	LRS	RCS
		T	0.065	0.051	0.009	0.106	Gm13_BLOCK338	MPW	a1	-0.018	0.583	0.693	0.441
Gm06_BLOCK201	MPW	a1	0.038	0.679	0.633	0.739			a2	-0.134	0.207	0.034	0.433
		a2	0.096	0.152	0.088	0.237			a3	-0.031	0.076	0.091	0.057
		a3	0.034	0.113	0.188	0.016			a4	-0.027	0.074	0.097	0.045
		a4	-0.168	0.055	0.091	0.008			a5	0.227	0.053	0.082	0.016
Gm06_BLOCK208	MPH	a1	0.198	0.434	0.426	0.445			a6	-0.017	0.005	0.003	0.008
		a2	0.144	0.262	0.138	0.424	Gm13_BLOCK396	MPH	a1	0.101	0.915	0.859	0.988
		a3	-0.013	0.190	0.257	0.102			a2	-0.101	0.085	0.141	0.012
		a4	-0.100	0.078	0.116	0.029	Gm14_3106285	MPH	T	-0.101	0.949	0.918	0.988
		a5	0.243	0.027	0.047	0.000			C	0.101	0.051	0.082	0.012
		a6	-0.124	0.007	0.013	0.000	Gm14_BLOCK408	MPH	a1	0.010	0.746	0.743	0.751
		a7	-0.347	0.002	0.003	0.000			a2	0.164	0.197	0.172	0.229
Gm06_34868214	MPH	G	-0.124	0.968	0.947	0.996			a3	0.235	0.027	0.047	0.000
		A	0.124	0.032	0.053	0.004			a4	0.170	0.021	0.022	0.020
Gm06_BLOCK401	MPW	a1	-0.059	0.622	0.520	0.755			a5	-0.588	0.007	0.013	0.000
		a2	-0.093	0.113	0.075	0.163			a6	0.009	0.002	0.003	0.000
		a3	0.082	0.090	0.138	0.029	Gm15_BLOCK93	MPW	a1	0.004	0.807	0.727	0.910
		a4	0.018	0.082	0.141	0.004			a2	-0.083	0.176	0.241	0.090
		a5	-0.004	0.046	0.060	0.029			a3	0.079	0.018	0.031	0.000
		a6	0.056	0.046	0.066	0.020	Gm15_BLOCK240	MPH	a1	-0.047	0.468	0.386	0.576
Gm06_BLOCK491	MPH	a1	0.050	0.514	0.417	0.641			a2	0.073	0.309	0.301	0.318
		a2	-0.175	0.193	0.266	0.098			a3	0.440	0.183	0.276	0.061
		a3	0.625	0.105	0.138	0.061			a4	-0.409	0.035	0.028	0.045
		a4	-0.147	0.069	0.025	0.127			a5	-0.056	0.005	0.009	0.000
		a5	-0.029	0.032	0.056	0.000	Gm15_30923425	MPW	C	0.081	0.972	1.000	0.935
		a6	0.242	0.027	0.003	0.057			T	-0.081	0.028	0.000	0.065
		a7	-0.498	0.020	0.025	0.012	Gm15_31242502	MPH	G	0.232	0.982	0.994	0.967
		a8	0.396	0.018	0.031	0.000			A	-0.232	0.018	0.006	0.033
		a9	-0.020	0.012	0.019	0.004	Gm15_BLOCK383	MPH	a1	0.179	0.599	0.539	0.678
		a10	-0.117	0.005	0.009	0.000			a2	0.514	0.115	0.172	0.041
		a11	-0.100	0.004	0.006	0.000			a3	-1.258	0.083	0.050	0.127
		a12	-0.227	0.002	0.003	0.000			a4	-0.225	0.076	0.110	0.033
Gm06_BLOCK522	MPW	a1	-0.172	0.589	0.690	0.457			a5	0.082	0.041	0.038	0.045
		a2	-0.082	0.241	0.122	0.396			a6	0.258	0.041	0.069	0.004
		a3	-0.170	0.117	0.157	0.065			a7	-0.183	0.035	0.006	0.073
		a4	-0.029	0.051	0.031	0.078			a8	0.149	0.007	0.013	0.000
		a5	0.454	0.002	0.000	0.004			a9	0.484	0.002	0.003	0.000
Gm07_BLOCK25	MPH	a1	0.065	0.332	0.219	0.478	Gm15_BLOCK409	MPW	a1	0.003	0.332	0.270	0.412
		a2	-0.050	0.275	0.317	0.220			a2	0.078	0.204	0.254	0.139
		a3	-0.062	0.257	0.326	0.167			a3	-0.015	0.105	0.157	0.037
		a4	0.047	0.137	0.138	0.135			a4	0.087	0.103	0.072	0.143
Gm07_3177189	MPW	T	-0.176	0.989	0.997	0.980			a5	0.115	0.076	0.075	0.078
		C	0.176	0.011	0.003	0.020			a6	0.080	0.064	0.110	0.004
Gm07_BLOCK272	MPH	a1	-0.139	0.977	0.966	0.992			a7	-0.070	0.044	0.047	0.041
		a2	0.139	0.023	0.034	0.008			a8	0.079	0.034	0.000	0.078
Gm07_BLOCK302	MPW	a1	-0.021	0.601	0.583	0.624			a9	-0.019	0.020	0.000	0.045
		a2	0.158	0.390	0.417	0.355			a10	-0.051	0.016	0.013	0.020
		a3	-0.121	0.007	0.000	0.016			a11	-0.333	0.002	0.003	0.000
		a4	-0.016	0.002	0.000	0.004			a12	0.045	0.002	0.000	0.004
Gm07_BLOCK373	MPW	a1	0.036	0.447	0.404	0.502	Gm16_2912151	MPW	C	-0.045	0.954	0.981	0.918
		a2	0.008	0.294	0.373	0.192			A	0.045	0.046	0.019	0.082
		a3	-0.044	0.259	0.223	0.306	Gm16_BLOCK67	MPH	a1	0.094	0.842	0.878	0.796
Gm07_42499533	MPH	G	-0.085	0.812	0.853	0.759			a2	-0.094	0.158	0.122	0.204
		A	0.085	0.188	0.147	0.241	Gm16_7534785	MPW	C	-0.074	0.954	0.978	0.922
Gm08_BLOCK71	MPW	a1	-0.007	0.931	0.878	1.000			T	0.074	0.046	0.022	0.078
		a2	-0.130	0.043	0.075	0.000	Gm16_BLOCK395	MPH	a1	0.061	0.516	0.580	0.433
		a3	-0.084	0.021	0.038	0.000			a2	-0.119	0.255	0.254	0.257
		a4	0.221	0.005	0.009	0.000			a3	0.057	0.229	0.166	0.310
Gm08_BLOCK106	MPH	a1	-0.161	0.348	0.270	0.449	Gm17_BLOCK344	MPW	a1	0.161	0.379	0.426	0.318
		a2	0.034	0.307	0.361	0.237			a2	0.177	0.337	0.361	0.306
		a3	-0.025	0.163	0.201	0.114			a3	0.123	0.271	0.191	0.376
		a4	0.033	0.106	0.097	0.118			a4	0.350	0.005	0.009	0.000
		a5	-0.143	0.055	0.034	0.082			a5	-0.214	0.004	0.006	0.000
		a6	0.262	0.021	0.038	0.000			a6	-0.539	0.002	0.003	0.000
Gm08_11056573	MPW	T	0.052	0.968	0.944	1.000			a7	-0.059	0.002	0.003	0.000

SNPLDB	Trait	Allele	Effect	CCSP	LRS	RCS	SNPLDB	Trait	Allele	Effect	CCSP	LRS	RCS
		A	-0.052	0.032	0.056	0.000	Gm18_BLOCK129	MPH	a1	0.002	0.727	0.790	0.645
Gm08_BLOCK165	MPW	a1	0.044	0.587	0.502	0.698			a2	-0.075	0.230	0.163	0.318
		a2	-0.044	0.413	0.498	0.302			a3	0.073	0.043	0.047	0.037
Gm08_BLOCK209	MPW	a1	-0.076	0.906	0.959	0.837	Gm18_BLOCK434	MPH	a1	-0.237	0.938	0.909	0.976
		a2	0.076	0.094	0.041	0.163			a2	-0.095	0.053	0.085	0.012
Gm08_BLOCK250	MPH	a1	-0.243	0.908	0.862	0.967			a3	0.332	0.009	0.006	0.012
		a2	-0.343	0.090	0.135	0.033	Gm18_BLOCK727	MPH	a1	0.001	0.569	0.589	0.543
		a3	0.586	0.002	0.003	0.000			a2	0.119	0.418	0.389	0.457
Gm08_28738663	MPH	G	-0.097	0.878	0.934	0.804			a3	-0.121	0.012	0.022	0.000
		A	0.097	0.122	0.066	0.196	Gm18_BLOCK729	MPH	a1	0.001	0.837	0.850	0.820
Gm08_BLOCK466	MPH	a1	0.074	0.551	0.467	0.661			a2	0.099	0.112	0.082	0.151
		a2	0.012	0.160	0.194	0.114			a3	-0.100	0.051	0.069	0.029
		a3	-0.089	0.080	0.122	0.024	Gm18_BLOCK736	MPW	a1	0.004	0.617	0.699	0.510
		a4	-0.022	0.073	0.038	0.118			a2	0.066	0.332	0.238	0.453
		a5	0.078	0.050	0.078	0.012			a3	-0.070	0.051	0.063	0.037
		a6	0.157	0.037	0.050	0.020	Gm19_37391411	MPH	T	0.170	0.989	0.981	1.000
		a7	0.320	0.025	0.006	0.049			C	-0.170	0.011	0.019	0.000
		a8	-0.362	0.012	0.022	0.000	Gm19_42142070	MPH	G	0.347	0.986	1.000	0.967
		a9	-0.168	0.012	0.022	0.000			A	-0.347	0.014	0.000	0.033
Gm09_2378279	MPW	G	-0.038	0.598	0.655	0.522	Gm19_42756453	MPH	T	-0.189	0.986	0.997	0.971
		A	0.038	0.402	0.345	0.478			G	0.189	0.014	0.003	0.029
Gm09_4538598	MPH	T	-0.045	0.504	0.448	0.576	Gm19_BLOCK554	MPW	a1	0.044	0.438	0.357	0.543
		C	0.045	0.496	0.552	0.424			a2	-0.019	0.314	0.367	0.245
Gm09_BLOCK115	MPW	a1	-0.005	0.309	0.263	0.367			a3	0.043	0.243	0.270	0.208
		a2	0.005	0.262	0.295	0.220			a4	0.061	0.004	0.006	0.000
		a3	0.024	0.151	0.085	0.237			a5	-0.129	0.002	0.000	0.004
		a4	-0.034	0.177	0.219	0.122	Gm20_BLOCK210	MPW	a1	-0.052	0.959	0.972	0.943
		a5	-0.047	0.082	0.113	0.041			a2	0.140	0.030	0.009	0.057
		a6	-0.103	0.016	0.019	0.012			a3	0.082	0.007	0.013	0.000
		a7	0.161	0.004	0.006	0.000			a4	-0.170	0.004	0.006	0.000
Gm09_BLOCK142	MPW	a1	-0.129	0.507	0.492	0.527	Gm20_BLOCK388	MPH	a1	-0.038	0.498	0.555	0.424
		a2	-0.111	0.443	0.483	0.392			a2	-0.051	0.328	0.335	0.318
		a3	-0.098	0.046	0.025	0.073			a3	0.142	0.103	0.044	0.180
		a4	0.338	0.004	0.000	0.008			a4	-0.053	0.071	0.066	0.078
Gm09_20590481	MPH	C	0.040	0.599	0.539	0.678	Gm20_BLOCK429	MPW	a1	0.046	0.933	0.909	0.963
		T	-0.040	0.401	0.461	0.322			a2	-0.046	0.067	0.091	0.037
Gm09_37359880	MPH	C	0.150	0.970	0.991	0.943	Gm20_39658098	MPH	G	0.164	0.973	1.000	0.939
		T	-0.150	0.030	0.009	0.057			A	-0.164	0.027	0.000	0.061
Gm10_BLOCK71	MPW	a1	0.015	0.539	0.520	0.563	Gm20_41737971	MPW	G	-0.034	0.934	0.893	0.988
		a2	-0.049	0.293	0.357	0.208			C	0.034	0.066	0.107	0.012
		a3	0.011	0.083	0.050	0.127	Gm20_BLOCK531	MPW	a1	0.081	0.594	0.602	0.584
		a4	0.071	0.073	0.060	0.090			a2	-0.018	0.275	0.301	0.241
		a5	-0.048	0.012	0.013	0.012			a3	0.040	0.083	0.063	0.110
Gm10_BLOCK96	MPW	a1	0.337	0.938	0.909	0.976			a4	-0.030	0.043	0.025	0.065
		a2	0.331	0.060	0.088	0.024			a5	-0.073	0.005	0.009	0.000
		a3	-0.668	0.002	0.003	0.000							

MPW and MPH represent the membership index values of relative plant weight and plant height, respectively. SNPLDB, SNP linkage disequilibrium block.

Table S4. The candidate genes of drought tolerance detected by qRT-PCR and their classifications.

Gene	SNPLDB	Trait	N23644 (T)		N00710 (S)		Supper	Classification								
			TL	TR	SL	SR		A	S	T	D	PM	TF	PK	UF	O
Glyma05g16373	Gm05_18374832	MPWMPH		30.6			√					√				
Glyma05g16380						6.39				√		√				
Glyma06g47300	**Gm06_BLOCK576 (RCS)**	MPW MPH	6.41		6.82				√	√						
Glyma08g40360	**Gm08_BLOCK466 (LRS)**	MPWMPH				5.3			√	√						
Glyma08g40420				5.24		9.71		√					√			
Glyma20g30980	Gm20_39658098 (RCS)	MPWMPH			0.16			√	√		√		√			
Glyma20g31015						6.45				√						
Glyma01g00321	**Gm01_149527**	MPW				7.31		√								
Glyma02g16170	**Gm02_14594196**	MPW	0.11												√	
Glyma02g16200				15		15.03		√	√	√						
Glyma02g16220				7.54		14.12										√
Glyma02g45940	**Gm02_BLOCK579 (LRS)**	MPW		8.03												√
Glyma03g01224	**Gm03_993540**	MPW		13.8		64.45		√	√			√				
Glyma03g01262			7.78	15.3		156.5	√	√	√			√				
Glyma03g21690	**Gm03_BLOCK283 (LRS)**	MPW			10.6			√	√		√	√				
Glyma06g21080				5.86		5.35										√
Glyma06g21090						13.93						√				
Glyma06g42121	**Gm06_BLOCK522 (RCS)**	MPW		9.61		20.46						√				
Glyma09g03350	**Gm09_2378279**	MPW				33.47					√	√				
Glyma10g06780	**Gm10_BLOCK71**	MPW		5.1									√			
Glyma10g06810			10.3					√	√		√					
Glyma10g11700	**Gm10_BLOCK159 (LRS)**	MPW	21.1													√
Glyma10g11706					5.35	10.41					√					
Glyma10g11720					9.58	28.64					√					
Glyma10g11775						0.11		√	√				√			
Glyma10g17230	**Gm10_BLOCK229 (LRS)**	MPW		5.94	9.65	69.55						√				
Glyma13g25290	**Gm13_BLOCK338**	MPW		0.02												√
Glyma13g25350			5.55	0.04				√	√							
Glyma13g25380				0.2	8.12								√			
Glyma13g25390				0.14						√	√					
Glyma13g25420				10.1				√	√							
Glyma13g40100	**Gm13_BLOCK486 (LRS)**	MPW	6.08					√	√	√						
Glyma13g40170					6.41	31.78						√				
Glyma13g40220				22.1												√
Glyma13g40240				11.2		7.73		√	√	√	√	√	√	√		
Glyma13g40251			5.31	10.7		10.93					√		√			
Glyma13g40260				26.6			√			√						
Glyma01g04151	Gm01_3646943	MPW		6.94		8.4				√						
Glyma01g04160						32				√						
Glyma01g04200				9.35		29.86		√	√							
Glyma02g08115	Gm02_BLOCK81	MPW	14.5		66.3		√	√	√	√						
Glyma02g08130				25	8.69	33.36	√			√						
Glyma02g08145				6		17.03		√					√			
Glyma02g08160				8.54						√						
Glyma02g08201				5.12		7.26										√
Glyma02g08210				5.92		8.63		√	√		√	√				
Glyma02g08231				8.49		71.01		√			√					
Glyma02g08241				13		11.96		√	√	√	√		√			
Glyma02g08250						39.95				√						
Glyma03g00590	Gm03_326463	MPW		6.66						√		√	√			
Glyma03g00980	Gm03_BLOCK11	MPW		8.66	9.06	7.46					√		√			
Glyma05g17470	Gm05_20554448	MPW			8.85	90.2	√		√							

Gene	SNPLDB	Trait	N23644 (T)		N00710 (S)		Supper	Classification								
			TL	TR	SL	SR		A	S	T	D	PM	TF	PK	UF	O
Glyma06g24540	Gm06_BLOCK264	MPW				10.16		√								
Glyma06g35630	Gm06_BLOCK401	MPW		5.8		54										√
Glyma07g21050	Gm07_BLOCK229 (LRS)	MPW				29.96										√
Glyma07g31880	Gm07_BLOCK373	MPW		8.85	7.24	20.04					√		√			
Glyma08g06870	Gm08_BLOCK49 (LRS)	MPW				5.19					√					
Glyma08g10070	Gm08_BLOCK71 (LRS)	MPW			6.04										√	
Glyma08g10080						48							√			
Glyma08g15250	Gm08_11056573 (LRS)	MPW				17.09				√						
Glyma08g45250	Gm08_BLOCK527 (LRS)	MPW		5.08		9.09							√			
Glyma09g09120	Gm09_BLOCK115 (LRS)	MPW		5.06		9.99									√	
Glyma09g09140						10.13									√	
Glyma09g09190						8.46									√	
Glyma09g11360	Gm09_BLOCK142 (RCS)	MPW	5.3	7.01		5.66				√						
Glyma09g28380	Gm09_35353845	MPW	0.18						√	√						
Glyma09g29760	Gm09_36608762	MPW			13.5	18.77					√		√			
Glyma10g08210	Gm10_BLOCK96 (LRS)	MPW				18		√								
Glyma10g29340	Gm10_38212261	MPW			7.01			√		√						
Glyma11g24236	Gm11_BLOCK241	MPW	0.13													√
Glyma11g27270	Gm11_26892595	MPW				16.45										√
Glyma12g10550	Gm12_8508827	MPW			7.67	25.11		√	√		√	√				
Glyma12g20460	Gm12_BLOCK254 (LRS)	MPW				5.28					√			√		
Glyma12g30780	Gm12_BLOCK402	MPW				7.84		√	√	√	√	√				
Glyma12g32950	Gm12_BLOCK429	MPW			7.57	50.91									√	
Glyma13g02310	Gm13_2179313	MPW				8.88		√					√			
Glyma14g32430	Gm14_39515432	MPW	22.3		96		√					√				
Glyma15g13420				36.9		42.67	√								√	
Glyma15g13430				23.7		5.74	√		√							
Glyma15g13440				8.72		6.36									√	
Glyma15g13450				10.5											√	
Glyma16g03450	Gm16_2912151	MPW		11.4		5.31									√	
Glyma16g08270	Gm16_7534785	MPW				19.56					√					
Glyma16g27350	Gm16_BLOCK365	MPW				11.47				√						
Glyma17g29840	Gm17_BLOCK344 (LRS)	MPW				5.43					√					
Glyma17g29850			5.46	10.3						√						
Glyma19g38890	Gm19_BLOCK554 (RCS LRS)	MPW		11.2	10.1	10.06						√		√		
Glyma20g05491	Gm20_6329124	MPW	5.58	11		36.25									√	
Glyma20g36520	Gm20_BLOCK531 (LRS)	MPW		5.24										√		
Glyma20g36540				8.51							√					
Glyma20g36550						5.35					√					
Glyma20g36580				10.8		36.25									√	
Glyma02g26160	**Gm02_BLOCK299 (LRS)**	MPH	9.35		22.9	81.01	√	√	√	√						
Glyma06g19220	**Gm06_BLOCK172**	MPH		15.6		98.02	√								√	
Glyma06g21540	**Gm06_BLOCK208 (LRS)**	MPH		6.68		19.49									√	
Glyma06g21560						10.74		√	√	√		√				
Glyma06g21570						83.58	√									
Glyma06g21584				10.5		133.9	√	√	√			√				
Glyma06g21596						57.88		√		√						
Glyma06g21608						35.38		√								
Glyma06g21620						10.16		√								
Glyma06g21651				5.86												√
Glyma06g21665					5.31	42.08		√	√	√						
Glyma06g21681						5.41					√					
Glyma06g21695						16.17		√								
Glyma06g21710						12.25		√								

Gene	SNPLDB	Trait	N23644 (T)		N00710 (S)		Supper	Classification								
			TL	TR	SL	SR		A	S	T	D	PM	TF	PK	UF	O
Glyma06g40170	**Gm06_BLOCK491 (LRS)**	MPH		6.87		127.6	√				√			√		
Glyma06g40240			5.05			48.84					√			√		
Glyma06g40270						6.45		√	√			√				
Glyma06g40291			5.92	8.17		24.59									√	
Glyma06g40311			5.92	8.4		58.08					√			√		
Glyma06g40330						11.63		√	√							
Glyma07g03770	**Gm07_BLOCK25**	MPH				7.41									√	
Glyma07g03780				9.99	8.69	32		√	√	√						
Glyma07g03790				13.6	9.51	47.5										√
Glyma07g03800						12.73									√	
Glyma08g14190	**Gm08_BLOCK106 (LRS)**	MPH				6.73									√	
Glyma08g14200						7.41										√
Glyma08g14210						0.06		√	√		√			√		
Glyma13g10680	**Gm13_BLOCK177 (LRS)**	MPH				28.05		√	√							
Glyma13g10690						61.82							√			
Glyma13g10720						13.83					√					
Glyma13g10750						6.23			√							
Glyma14g26830	**Gm14_BLOCK408 (LRS)**	MPH	22.3		96		√								√	
Glyma14g26960					5.46	132.5	√							√		
Glyma14g27015					5.17	76.11								√		
Glyma15g30265	**Gm15_BLOCK383 (LRS)**	MPH				8.63					√					
Glyma16g32170	**Gm16_BLOCK395**	MPH	0.2							√						
Glyma16g32180			0.11							√						
Glyma01g34780	Gm01_BLOCK493 (RCS)	MPH	0.13					√			√	√		√		
Glyma01g34800				18.1				√	√							
Glyma01g34830				5.33						√	√	√				
Glyma01g39120	Gm01_BLOCK546	MPH				30.7									√	
Glyma01g39140						18.64									√	
Glyma02g00371	Gm02_161337	MPH		7.97											√	
Glyma02g46800	Gm02_50652770	MPH				5.43				√						
Glyma04g33410	Gm04_BLOCK490 (RCS)	MPH				5.43					√		√			
Glyma04g33540						81.57	√								√	
Glyma04g41740	Gm04_47582011	MPH			12.5					√	√		√			
Glyma06g33850	Gm06_34868214	MPH		7.89		6.7		√	√							
Glyma07g16651	Gm07_16348924	MPH		12.1	17.5	89.57	√								√	
Glyma07g16660				6.23												√
Glyma07g16690						45.1					√					
Glyma07g18280	Gm07_BLOCK194	MPH	16.6		75.6	211.6	√	√	√							
Glyma07g28770	Gm07_32919498	MPH		6.06	14.9	75.32									√	
Glyma07g37440	Gm07_42499533	MPH		7.16		12.77										√
Glyma09g05781	Gm09_4538598	MPH		9.45	20.3	5.94							√			
Glyma09g05800				5.35	11.6	10.2									√	
Glyma09g16901	Gm09_20590481	MPH		11.8	7.81	24.93						√				
Glyma09g35990	Gm09_41866356	MPH			0.15										√	
Glyma11g16653	Gm11_BLOCK135 (LRS)	MPH				12.82										√
Glyma11g16700				21.6											√	
Glyma11g16750			32.8			8	√	√	√							
Glyma11g27920	Gm11_27967762	MPH			23										√	
Glyma11g29770	Gm11_BLOCK344	MPH		14.7											√	
Glyma11g29800						9.78				√	√	√				
Glyma11g29810				16.3		56.1										√
Glyma12g29190	Gm12_32591630	MPH				45.25		√		√						
Glyma13g19830	Gm13_23309035	MPH		7.57		6.36										√
Glyma13g25200	Gm13_28457573	MPH		15.7	13.3										√	

Gene	SNPLDB	Trait	N23644 (T)		N00710 (S)		Supper	Classification								
			TL	TR	SL	SR		A	S	T	D	PM	TF	PK	UF	O
Glyma13g25240				20.8	33.2					√	√					
Glyma13g30090	Gm13_BLOCK396	MPH		9	31.5	18.64		√	√		√	√		√		
Glyma13g30100						18						√		√		
Glyma13g30110				10.3	22.9			√				√		√		
Glyma14g21901	Gm14_25589678	MPH				6.92		√	√	√				√		
Glyma16g06770	Gm16_BLOCK67	MPH		108			√									√
Glyma18g10830	Gm18_BLOCK129	MPH			34.7										√	
Glyma18g10850					0.09											√
Glyma18g33230	Gm18_BLOCK434	MPH				7.41									√	
Glyma18g52360	Gm18_BLOCK727 (LRS)	MPH		12.3	11.4					√						
Glyma18g52430	Gm18_BLOCK729	MPH			0.16											√
Glyma18g52470					32.6		√								√	
Glyma19g33820	Gm19_41440516	MPH		5.88		7.57									√	
Glyma19g34540	Gm19_42142070 (RCS)	MPH		0.15	6.52								√			
Glyma19g35236	Gm19_42756453	MPH			11	5.62		√	√	√						
Glyma19g35251					24.9	18		√	√	√						
Glyma19g35261				18.2				√	√	√						

Two accessions, drought tolerant N23644 (T) and drought sensitive N00710 (S), with MPWs of 0.750 and 0.098, respectively, were used for differential expression analysis using qRT-PCR. Here listed are two relative values (treated vs respective control) of leaf (L) and root (R) for the 177 genes displaying a significantly differential expression in response to 1.0 h-10% PEG in at least one pair of comparison. The candidate genes and corresponding QTL in boldface represents the gene is located in a large-contribution major QTL. In the columns of SNPLDB, the "LRS" in the parentheses represent the locus carrying allele(s) specific to the landrace subpopulation; the "RCS" in the parentheses represent the locus carrying allele(s) specific to the released cultivar subpopulation. In the Trait column, MPW and MPH represent the membership index values of relative plant weight and plant height, respectively. Supper: the supper candidate genes with the relative expression value more than 1.5 times the inter-quartile range based on boxplot are marked with "√". In Classification column, A, S, T, D, PM, TF, PK, UF and O, represent the nine biological categories of candidate genes, i.e. ABA responders, stress responders, transports, development factors, protein metabolism, transcription factors, protein kinases, unknown function and others, respectively.

Table S5. The candidate gene-allele system of DT in soybean.

Gene	Trait	P	Allele	Effect	Gene	Trait	P	Allele	Effect
Glyma01g00321	MPW	0.278	G	0.418	*Glyma04g33410*	MPH	0.574	A	0.123
			A	0.509				G	0.081
Glyma01g04160	MPW	0.344	A	0.437	*Glyma04g41740*	MPH	0.559	C	0.122
			G	0.414				T	0.056
Glyma02g08115	MPW	0.043	AC	0.440	*Glyma06g33850*	MPH	0.400	G	0.123
			GT	0.398				A	0.041
Glyma02g16220	MPW	0.611	A	0.292	*Glyma06g40240*	MPH	0.015	TTT	0.158
			T	0.420				TTC	0.071
Glyma03g00590	MPW	0.349	CC	0.431				TAT	0.113
			AC	0.399				ATT	-0.046
			CA	0.399	*Glyma06g40330*	MPH	0.253	T	0.136
Glyma03g01262	MPW	0.043	T	0.429				C	0.094
			C	0.373	*Glyma07g03770*	MPH	0.016	TA	0.085
Glyma05g16380	MPW	0.555	A	0.419				GG	0.171
			C	0.567	*Glyma07g03780*	MPH	0.983	A	0.121
	MPH	0.963	A	0.121				T	0.120
			C	0.101	*Glyma07g16651*	MPH	0.962	T	0.121
Glyma05g17470	MPW	0.555	C	0.419				C	0.114
			T	0.567	*Glyma07g37440*	MPH	0.002	T	0.099
Glyma06g24540	MPW	0.707	C	0.419				G	0.259
			T	0.474	*Glyma08g14200*	MPH	0.009	A	0.154
Glyma06g35630	MPW	0.826	TG	0.419				G	0.057
			CG	0.426	*Glyma09g35990*	MPH	0.454	CG	0.135
			CA	0.393				TG	0.103
Glyma06g47300	MPW	0.587	A	0.412				CT	0.000
			T	0.424	*Glyma11g16653*	MPH	0.138	G	0.097
	MPH	0.702	A	0.112				C	0.150
			T	0.126	*Glyma13g10680*	MPH	0.159	A	0.055
Glyma08g15250	MPW	0.056	TC	0.406				G	0.130
			TG	0.437	*Glyma13g10720*	MPH	0.825	C	0.123
			AG	0.535				G	0.115
Glyma09g09140	MPW	0.867	A	0.419	*Glyma13g10750*	MPH	0.306	ACACCC	0.122
			G	0.425				ACACCT	0.145
Glyma10g29340	MPW	0.009	A	0.353				ATACCT	0.051
			G	0.431				GCGTCT	0.198
Glyma12g30780	MPW	0.233	C	0.415				GCGTTT	-0.013
			T	0.454	*Glyma13g25200*	MPH	0.177	CATTCGC	0.111
Glyma12g32950	MPW	0.000	CGC	0.383				CATTTAT	0.116
			GGC	0.463				AATTTAT	0.114
			CCT	0.476				CACTCGC	0.145
Glyma10g11700	MPW	0.336	TA	0.418				CCTTCGC	0.056
			CG	0.475				CATGCGC	0.360
			TG	0.407	*Glyma13g30090*	MPH	0.139	TC	0.114
Glyma10g11720	MPW	0.269	GGC	0.418				GC	0.324
			AGT	0.48				TT	0.278
			GTC	0.408	*Glyma15g30265*	MPH	0.711	GCCCT	0.120
Glyma16g03450	MPW	0.456	CTC	0.416				AACTT	0.108
			CCT	0.492				GCACT	0.175
			ACC	0.459				GCCCC	0.055
Glyma16g27350	MPW	0.004	CG	0.428	*Glyma16g32180*	MPH	0.010	TG	0.171
			TA	0.316				GG	0.071
Glyma20g36580	MPW	0.484	T	0.420				GC	-0.025
			G	0.369	*Glyma18g52430*	MPH	0.849	GG	0.123
Glyma01g34780	MPH	0.813	AC	0.121				AA	0.090
			GT	0.101				AG	0.075
Glyma01g39120	MPH	0.307	A	0.128	*Glyma19g35236*	MPH	0.124	C	0.124
			T	0.077				A	-0.106
Glyma02g00371	MPH	0.587	AG	0.116					
			GG	0.156					
			GA	0.100					

Table S6. The GO functional classification of the candidate DT genes related to the two indicators.

Class	A	S	T	D	PM	TF	PK	UF	O	No.
Whole	51(0.29)	41(0.23)	41(0.23)	38(0.21)	26(0.15)	21(0.12)	15(0.08)	35(0.2)	22(0.12)	177
MPW	25(0.27)	22(0.24)	24(0.26)	23(0.25)	17(0.18)	16(0.17)	4(0.04)	12(0.13)	11(0.12)	92
MPH	28(0.30)	22(0.24)	21(0.23)	16(0.17)	11(0.12)	7(0.08)	11(0.12)	23(0.25)	11(0.12)	92
Shared	2(0.29)	3(0.43)	4(0.57)	1(0.14)	2(0.29)	2(0.29)	0(0.00)	0(0.00)	0(0.00)	7
S-QTL-allele	25(0.29)	25(0.26)	18(0.19)	27(0.28)	14(0.15)	12(0.13)	16(0.17)	14(0.15)	10(0.11)	95

The data showed that the detected QTL/SNPLDB shared by two or more indicators has an annotated gene frequency distribution among the biological function categories similar to the distribution of the total annotated genes. It means all the biological function categories being necessary for drought tolerance. A, S, T, D, PM, TF, PK, UF and O, represent the nine biological categories of candidate genes, i.e. ABA responders, stress responders, transports, development factors, protein metabolism, transcription factors, protein kinases, unknown function and others, respectively. The number outside of the parentheses is the total number of genes, and the number in parentheses is its rate to the total number of genes. S-QTL-allele, the gene in the 58 deficient and 16 emerged QTL-alleles changed from LRS to RCS.

Table S7. Comparisons between DT mapping results from the present RTM-GWAS and those reported from linkage mapping.

SNPLDB	Position (bp)	R^2	Reported QTL	Position (bp)	Population
Gm02_14594196	14594196	1.0	RRL2.5	15084508-15269219	5
Gm06_BLOCK172	15431276-15435935	1.0	RRL6.3	16450669-16456975	5
Gm06_BLOCK201	17499686-17547717	1.5	RRL6.4	17458582-17605275	5
Gm16_BLOCK395	35357235-35358822	1.6	WUE 1-4	36641646-37139174	2
Gm10_BLOCK159	12053290-12110973	2.5	Drought index 1-10	11305953-12771348	1
			Drought tolerance 6-3	12171935-43451323	3
Gm20_BLOCK388	33593168-33630697	1.0	RRL20.3	33980711	5
Gm20_BLOCK429	36186946-36187233	1.0	RRL20.4	35376980-35573994	5
Gm01_BLOCK493	47264933-47293823	0.9	Drought index 1-1	47892575-48314028	1
			RSL1.1	47679785-47719340	5
Gm02_161337	161337	0.4	RRL2.1	475383-665256	5
Gm03_326463	326463	0.4	RSL3.1	345540-391133	5
Gm04_BLOCK490	39059520-39224224	0.8	RRL4.3	39024528-39224011	5
Gm05_10333534	10333534	0.3	Drought tolerance 6-2	2557874-32388406	3
Gm05_18374832	18374832	0.4	Drought tolerance 6-2	2557874-32388406	3
Gm05_20554448	20554448	0.4	Drought tolerance 6-2	2557874-32388406	3
Gm05_33077723	33077723	0.2	Drought index 1-7	34163842-35266095	1
Gm06_BLOCK264	22103834-22103856	0.4	RRL6.5	22700125-22889619	5
Gm06_34868214	34868214	0.3	Drought index 1-2	27940564-35215132	1
Gm06_BLOCK522	45336128-45364730	0.8	RSL6.2	44421459	5
Gm07_3177189	3177189	0.7	RSL7.1	3637584-3637602	5
Gm07_BLOCK272	26276821-26276947	0.5	RSL7.2	26650890-26840665	5
Gm07_BLOCK373	36845443-36846591	0.3	RRL7.1	37156574-37328831	5
Gm07_42499533	42499533	0.5	Drought index 1-6	40968705-43225732	1
Gm08_BLOCK165	16183060-16183086	0.5	RSL8.2	16514519	5
Gm08_BLOCK527	44688033-44689115	0.7	RRL8.5	44810051-44999995	5
Gm09_BLOCK115	8621107-8819963	0.9	RRL9.1	8307615-8338394	5
Gm09_BLOCK142	11337674-11393438	0.7	RSL9.2	11146429-11345432	5
Gm09_41866356	41866356	0.2	RSL9.3	42125408-42323074	5
Gm12_BLOCK402	34418573-34433816	0.5	Drought index 1-3	35107930-36042990	1
Gm12_BLOCK429	36325875-36326233	0.8	Drought tolerance 6-4	34693738-36615077	3
			RRL12.1	36587265	5
Gm14_3106285	3106285	0.2	RSL14.1	4132705-4327965	5
Gm16_2912151	2912151	0.2	Drought index 1-5	1140933-1631658	1
Gm18_26517331	26517331	0.3	WUE 1-1	21357362-45919323	2
Gm18_BLOCK434	39409732-39410305	0.4	WUE 1-1	21357362-45919323	2
Gm19_37391411	37391411	0.2	RRL19.4	36520851-36592067	5
Gm19_45043655	45043655	0.2	RSL19.5	44377195-44558289	5
Gm20_BLOCK468	38753573-38753583	0.2	Drought index 1-4	37076339-38389840	1
Gm20_BLOCK531	44594152-44673992	0.6	RSL20.2	43939431-44098961	5
Gm04_47582011	47582011	0.6	WUE 2-2	43566128-44182293	4
			WUE 2-3	43566128-44182293	4
Gm19_46882319	46882319	0.3	WUE 2-1	47049000-47258164	4

Reported: 1, 2, 3, 4 and 5 represent a SNPLBD located in the interval of a QTL detected in the populations of 'Kefeng 1 × Nannong 1138-2'[1], 'Young × PI 416937'[2], 'Benning × PI 416937'[3], 'S100 × Tokyo'[4] and a nested association mapping (NAM) population[5] composed of 'M8206 × TongShan' and 'ZhengYang × M8206', respectively, which were reported in the literature.

Table S8. The 19 important and specific QTL/SNPLDB worth for further study.

	SNPLDB	Candidate Gene	Classification								
			ABA	Stress	T	D	PM	TF	PK	UF	O
**Two shared LC-major QTL**											
1	Gm06_BLOCK576	*Glyma06g47300*		√	√						
2	Gm08_BLOCK466	***Glyma08g40360***		√	√						
		Glyma08g40420	√					√			
		Glyma10g11720				√					
		Glyma10g11775	√	√				√			
**Two most significant QTL for each indicators**											
3	Gm13_BLOCK338	*Glyma13g25290*									√
		Glyma13g25350	√	√							
		Glyma13g25380						√			
		Glyma13g25390			√	√					
		Glyma13g25420	√	√							
4	Gm07_BLOCK25	*Glyma07g03770*								√	
		Glyma07g03780	√	√	√						
		Glyma07g03790									√
		Glyma07g03800								√	
**Five LC-major QTL repeatedly detected by linkage mapping**											
5	Gm02_14594196	***Glyma02g16170***								√	
		Glyma02g16181								√	
		Glyma02g16200	√	√	√						
		Glyma02g16220									√
6	Gm06_BLOCK172	***Glyma06g19220***								√	
7	Gm06_BLOCK201	***Glyma06g21050***								√	
		Glyma06g21080									√
		Glyma06g21090					√				
8	Gm16_BLOCK395	*Glyma16g32170*		√							
		Glyma16g32180			√						
9	Gm10_BLOCK159	*Glyma10g11700*								√	
		Glyma10g11706				√					
		Glyma10g11720				√					
		Glyma10g11775	√	√				√			
**Three QTL with the strongest differentiation between the LRS and RCS**											
10	Gm11_BLOCK241	Glyma11g24236									√
3	Gm13_BLOCK338										
**Twenty-four QTL with new allele emerged in RCS**											
11	Gm01_BLOCK493	*Glyma01g34780*	√			√	√		√		
		Glyma01g34800	√	√							
		Glyma01g34830			√	√	√				
12	Gm04_BLOCK232	*Glyma04g16805*		√							
13	Gm04_BLOCK490	*Glyma04g33410*				√		√			
		Glyma04g33540								√	
14	Gm06_BLOCK522	*Glyma06g42121*					√				
1	Gm06_BLOCK576	*Glyma06g47300*		√	√						
15	Gm09_BLOCK142	*Glyma09g11360*			√						
16	Gm15_BLOCK409	*Glyma15g32800*		√			√		√		
17	Gm19_42142070	*Glyma19g34540*				√					
18	Gm19_BLOCK554	*Glyma19g38890*					√		√		
19	Gm20_39658098	*Glyma20g30980*	√	√		√		√			
		Glyma20g31015			√						

T, D, PM, TF, PK, UF and O, represent the seven biological categories of candidate genes, i.e. transports, development factors, protein metabolism, transcription factors, protein kinases, unknown function and others, respectively; LRS, landrace subpopulation; RCS released cultivar subpopulation.

Table S9. Comparison of the thorough RTM-GWAS results with these only for some major ones with conservative significance level.

Result	RTM-GWAS for Entire QTL	Regular Conservative GWAS for Major QTL
Number of QTL detected	135	29
Total contribution R^2	≈50.9%	≈25.3%
Number of shared QTL	4	1
Number of Alleles detected	454	159
Number of RT-qPCR-verified genes	177	69
